# Genes, pathways and transcription factors involved in seedling stage chilling stress tolerance in *indica* rice through RNA-Seq analysis

**DOI:** 10.1186/s12870-019-1922-8

**Published:** 2019-08-14

**Authors:** Sharat Kumar Pradhan, Elssa Pandit, Deepak Kumar Nayak, Lambodar Behera, Trilochan Mohapatra

**Affiliations:** 1 Crop Improvement Division, National Rice Research Institute, Cuttack, Odisha India; 2 Indian Council of Agricultural Research, New Delhi, India

**Keywords:** Chilling stress tolerance transcripts, Rice cold stress transcriptome analysis, RNA-seq seedling stage cold tolerance, Cold-transcription factor genes, Gene ontology, KEGG pathway analysis

## Abstract

**Background:**

Rice plants show yellowing, stunting, withering, reduced tillering and utimately low productivity in susceptible varieties under low temperature stress. Comparative transcriptome analysis was performed to identify novel transcripts, gain new insights into different gene expression and pathways involved in cold tolerance in rice.

**Results:**

Comparative transcriptome analyses of 5 treatments based on chilling stress exposure revealed more down regulated genes in susceptible and higher up regulated genes in tolerant genotypes. A total of 13930 and 10599 differentially expressed genes (DEGs) were detected in cold susceptible variety (CSV) and cold tolerant variety (CTV), respectively. A continuous increase in DEGs at 6, 12, 24 and 48 h exposure of cold stress was detected in both the genotypes. Gene ontology (GO) analysis revealed 18 CSV and 28 CTV term significantly involved in molecular function, cellular component and biological process. GO classification showed a significant role of transcription regulation, oxygen, lipid binding, catalytic and hydrolase activity for tolerance response. Absence of photosynthesis related genes, storage products like starch and synthesis of other classes of molecules like fatty acids and terpenes during the stress were noticed in susceptible genotype. However, biological regulations, generation of precursor metabolites, signal transduction, photosynthesis, regulation of cellular process, energy and carbohydrate metabolism were seen in tolerant genotype during the stress. KEGG pathway annotation revealed more number of genes regulating different pathways resulting in more tolerant. During early response phase, 24 and 11 DEGs were enriched in CTV and CSV, respectively in energy metabolism pathways. Among the 1583 DEG transcription factors (TF) genes, 69 WRKY, 46 bZIP, 41 NAC, 40 ERF, 31/14 MYB/MYB-related, 22 bHLH, 17 Nin-like 7 HSF and 4C3H were involved during early response phase. Late response phase showed 30 bHLH, 65 NAC, 30 ERF, 26/20 MYB/MYB-related, 11 C3H, 12 HSF, 86 Nin-like, 41 AP2/ERF, 55 bZIP and 98 WRKY members TF genes. The recovery phase included 18 bHLH, 50 NAC, 31 ERF, 24/13 MYB/MYB-related, 4 C3H, 4 HSF, 14 Nin-like, 31 bZIP and 114 WRKY TF genes.

**Conclusions:**

Transcriptome analysis of contrasting genotypes for cold tolerance detected the genes, pathways and transcription factors involved in the stress tolerance.

**Electronic supplementary material:**

The online version of this article (10.1186/s12870-019-1922-8) contains supplementary material, which is available to authorized users.

## Background

Dependence of more than 50% of world population on rice for carbohydrate source makes it one of the most important food crops. Rice is cultivated in a wide range of ecology starting from upland to deepwater ecology including varying altitudes, climate and diverse soil types. Abiotic stresses like cold, high temperature, drought, salinity and flooding affect adversely in rice production. Rice being chilling-sensitive plant shows yellowing, slow seedling growth, stunting, withering, reduced tillering and utimately low productivity in cold susceptible varieties under low temperature stress [1–4]. Globally, about 15 million hectares of rice fields in 24 countries are affected by cold weather. Cultivation of rice is not possible in approximately 7 million hectares of land in south and south east Asia due to cold stress [5]. Now-a-days, the extent of unexpected cold weather has increased due to extreme weather events. The yield of rice in Australia, Korea, India, China, Japan and many other countries is affected by cold stress [2, 3, 6]. The dry season rice of India particularly *boro* rice faces cold stress during seedling stage. The stress covers around 4 million hectares of rice areas of the country targeting the seedling-stage causing delay in growth of the plant which ultimately coincide with high temperature stress during flowering stage [3, 7]. Therefore, developing high yielding varieties possessing cold stress tolerance may help in further augmenting rice production in these vulnerable regions. Besides, more insight into the molecular mechanisms of tolerance in response to chilling stress need to be revealed.

Plants adopt different strategies to escape from stresses based on their occurrence, severity and ecology [8]. Stresses faced by plants in response to wounding, pathogen attack, UV exposure, flood, drought, salt, heat and cold stress and combination of these stresses enhances production of reactive oxygen species (ROS) levels [9–12]. ROS homeostasis is observed to regulate strongly the cold responsive genes in arabidopsis. Earlier research results revealed the role of cold stress binding factors (CBFs) in ROS detoxification [13, 14]. Besides, ROS-mediated regulatory module that functions as an early component of the chilling stress response pathway is reported in rice [15]. Reactive oxygen species may be taken as a point of convergence for various gene networks in response to stress situation [16]. Transcription factors (TFs) are the master regulators for controlling expression of many target genes in a net work mode of single TF of down-stream genes. Earlier results suggest the role of AREB/ABF, NAC regulons and DREB1/ CBF in regulating salinity, temperature and drought stress responsive gene expression in rice [16, 17]. Reports of heat shock factor (HSFs) genes have been suggested as an important node of cross-talk in rice [18]. Research findings of a common set of TFs working in convergence with stress signaling networks and regulons of the upstream regulatory genes for controlling plant responses to multiple stresses [19]. A network mode of functioning of target TFs for similar molecular, biochemical, genetical and physiological processes are performed in response to stresses. A structural basis for the generation of unique patterns of gene expression is provided by binding of the TFs in combinations with cis-element. In a recent cloning study of COLD1 quantitative trait locus revealed mechanism of chilling tolerance in which the QTL interact with alpha sub-unit of G-protein for activating the Ca^2+^ channel which enhance the GTPase activity of G-protein for expressing chilling tolerance in *japonica* rice [20]. However, the mechanisms underlying rice chilling tolerance is still not fully known.

Many researchers previously studied rice transcriptome using microarrays [20–25]. In recent years, researchers used RNA-seq technology to profile and compare the transcriptome and understand molecular pathways in rice [26–31]. The RNA-seq technology has true advantage of representing the global transcriptome, which is limited in microarray since it depends on predefined probes. Rice genotype, Geetanjali was categorized as very highly tolerant and Sahabhagidhan as highly susceptible type to chilling stress reported by earlier publications [2–4]. The highly tolerant genotype Geetanjali could survive upto 25 days under chilling stress of 4 °C. Therefore, in the present investigation, we have compared the transcriptome of contrasting genotypes for chilling stress tolerance to identify novel transcripts, gain new insights into different gene expression and pathways involved in cold tolerance in rice.

## Results

### Phenotyping of contrasting genotypes for chilling stress tolerance

Upon exposure to low temperature stress, the susceptible genotype, Shahabhagidhan turned pale green and rolled leaves exhibiting a score of 5 after 7 days of exposure at 15 °C; whereas the resistant genotype, Geetanjali remained normal in growth and leaf coloration. At 4 °C, Sahabhagidhan had rolled leaf with yellow to brown in color and reduced growth showing a score of 7 while tolerant genotype, Geetanjali was normal. After 7 days of chilling at 4 °C, susceptible genotype, Sahabhagidhan died showing a SES score of 9 while Geetanjali was highly tolerant with a score of 1. The tolerant genotype showed scores of 5, 7 and 9 at chilling stress of 4 °C on 15th, 20th and 25th day, respectively.

### RNA sequencing and differentially expressed genes in response to chilling stress

RNA-seq generated 566 million raw reads in which 431 million high quality reads (QV > 25) with an average of 36 million reads per sample were obtained (Additional file 7: Table S1). High quality reads were mapped to the rice reference genome of cv. Nipponbare (*Oryza sativa L. subsp*. *japonica*) ranging from 92.4–95.1% per sample.

**Fig. 1:**
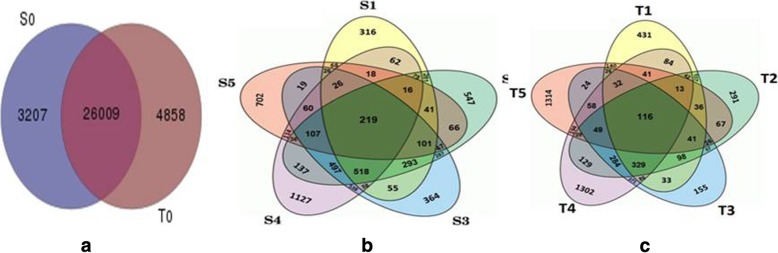
Venn diagram of differentially expressed genes (DEGs) of (**a**) two genotypes, susceptible (CSV) and tolerant (CTV) under room temperature (S_0_ and T_0_) (**b**) CSV and CTV cultivars under cold stress (6–48 h) and 48 h recovery conditions. S_1_-S_5_ and T_1_-T_5_ denote CSV and CTV at 6, 12, 24, 48 and 24 h recovery after 48 h stress conditions, respectively

Differentially expressed genes for chilling stress and subsequent recovery conditions were identified using fold change (FC) greater than 0 and significant in ‘t’ test (*p* < 0.05). We detected a total of 24529 DEGs in both the genotypes of which 13930 and 10599 DEGs were observed in CSV and CTV, respectively (Fig. 1). Out of these, 1666 and 2206 DEGs were found in subsequent recovery condition of CSV and CTV, respectively (Fig. 1). Furthermore, there was significant increase in DEGs detected in both the genotypes at 6, 12, 24 and 48 h exposure of cold stress with FC value > 4. To assess similarities and differences between cold contrasting genotypes, hierarchical cluster analysis using CuumeRbund was performed. The down-regulated gene populations in CSV was more than up-regulated genes while reverse was observed in CTV (Table 2). Further 12, 24 and 48 h cold stressed samples of both genotypes cluster together, but at 6 h cold stressed samples of both genotypes formed separate cluster. In addition, 24 h recovery samples of these two genotypes were grouped into a single cluster (Additional file 1: Figure S1). The comparative profile of differentially expressed genes in both the genotypes showed that more numbers of DEGs were down-regulated as compared to up-regulated genes except 48 h recovery condition (Additional file 8: Table S2; Additional file 2: Figure S2). Genes up-regulation and down-regulation in CSV above FC value was 24.97 and 75.03%, respectively. But in CTV, 43.92 and 56.08% genes were detected to be up-regulated and down-regulated, respectively (Additional file 2: Figure S2; Additional file 9: Table S3; Additional file 10: Table S4; Additional file 11: Table S5).

### Functional classification of DEGs

**Fig. 2:**
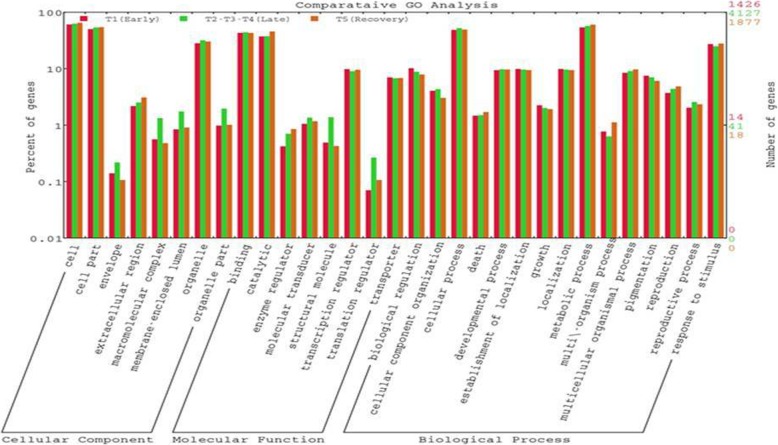
GO analysis of DEGs in early, late and 24 h recovery stages of tolerant cultivar, Geetanjali

**Fig. 3:**
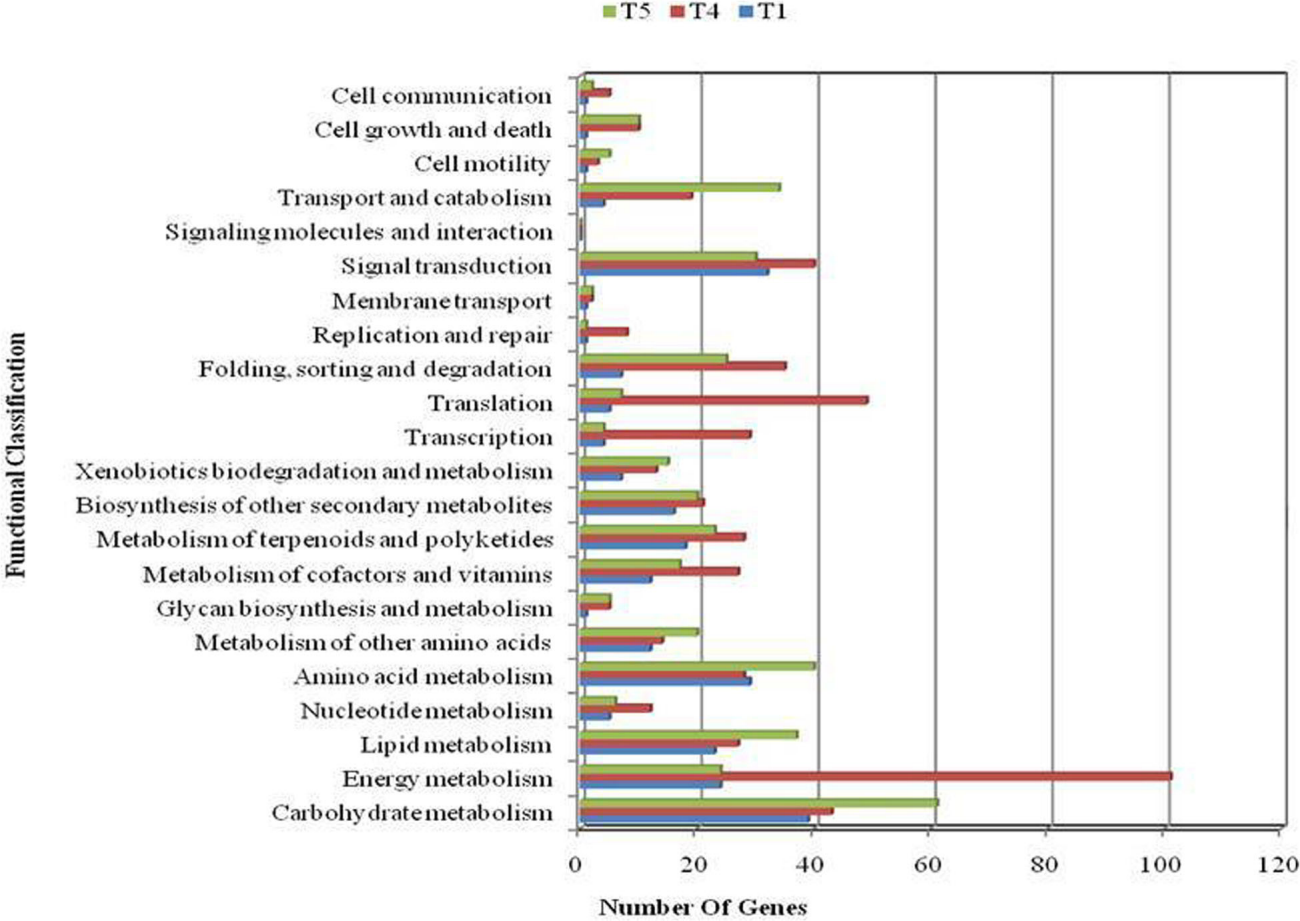
KEGG pathway incident of DEGs of CTV genotype at 6 h early response phase (T1), 48 h late response phase (T4) and 24 h recovery (T5)

Out of total 24529 DEGs, 14632 DEGs were GO term enriched in which 8047 DEGs observed in CSV and 6585 DEGs in CTV of which at least one term of GO classification categorized as molecular function, cellular component and biological process. Among all, 18 and 28 GO terms were significantly involved in molecular function, cellular component and biological process of CSV and CTV, respectively (Figs. 2 and 3).

Under molecular function category, five GO terms (GO:0003700; GO: GO:0003677; GO:0003824; GO:0016787; GO:0008289) were enriched and played a significant role in transcription regulation, oxygen, DNA and lipid binding, catalytic and hydrolase activity at any of three phases of CSV while nine GO terms (GO:0019825; GO:0030246; GO:0016740; GO:0003700; GO:0016301; GO:0016772; GO:0003824; GO:0005215; GO:0016787) were enriched in CTV. Both the genotypes showed 3 common GO terms enrichment during the cold stress. Catalytic activity, hydrolase activity and lipid binding molecular function were not enriched during the stress period in susceptible genotype. The cellular component category was enriched with six GO terms (GO: 0030312: GO: 0019825; 0005618; GO: 0005576; GO: 0009579; GO: 0009536) of which 3 GO terms (GO: 0030312; GO: 0005618; GO: 0005576) were significantly involved in all three phase of CSV genotype while in CTV, these were enriched in 24 h recovery condition. The GO term GO: 0009536 was enriched in 24 h recovery in CSV. The biological process category was enriched with 7 and 15 GO terms in at least any of the phases of CSV and CTV, respectively. Amongst these, 6 GO terms namely secondary metabolic process, response to stimulus, response to endogenous stimulus, response to stress, response to abiotic stimulus and response to biotic stimulus were enriched in both the genotypes. Examination of lipid metabolic process showed the same biological function in both susceptible and tolerant genotypes while signal transduction, regulation of cellular process, pollen-pistil interaction, regulation of biological process, photosynthesis, generation of precursor metabolites and energy, carbohydrate metabolic process were detected and enriched in early response phase of CTV (GO: 0007165; GO: 0050794; GO: 0065007; GO: 0009875; GO: 0050789; GO: 0015979; GO: 0006091; GO: 0005975). Further, DEGs in all the three response phases were described on the basis of GO term enrichment. In early response phase, the DEGs in biological process, molecular function and cellular component were detected in response to cold stress (Additional file 11: Table S5; Additional file 17: Table S11). It was highly surprising that no DEGs of CTV enriched for cellular component, whereas 102 DEGs of CSV were represented mainly for cell wall and all of them were down regulated. Similarly, the DEGs obtained for 3 classes of GO categorization in late response phase enriched due to cold stress (Additional file 12: Table S6; Additional file 15: Table S9; Additional file 18: Table S12). In recovery phase, CSV showed DEGs in 6 biological categories (secondary metabolic process, response to stimulus, response to abiotic stimulus, lipid metabolic process, response to endogenous stimulus, and response to stress) and no DEGs represented for transcription factor activity/regulation. In CTV, biological process DEGs were represented in 8 functional categories, out of which response to stimulus and stress, metabolic process, lipid and carbohydrate metabolism were affected (Additional file 13: Table S7; Additional file 16: Table S10).

### Kyoto encyclopedia of genes and genomes (KEGG) pathway annotation

KEGG pathway database was used to identify cold stress related genes involved in the pathways using DEGs. It was found that cold treatment continuously increased the number of up-regulated and down-regulated genes in both CSV and CTV genotypes, while it decreased after 24 h recovery, except exclusively expressed genes of CTV during recovery (Table 2; Additional file 2: Figure S2). Comparative analysis of two genotypes under control condition revealed 29216 and 30867 genes expressed in susceptible and tolerant genotypes, respectively.

The commonly expressed genes (26009) in both genotypes may be involved in plant growth and development. In early response phase, out of 2173 DEGs, 297 DEGs were enriched in KEGG pathway in CSV while 243 DEGs were enriched in CTV from 1655 DEGs (Fig. 3: Additional file 5: Figure S5). Comparison of early response phase in both the genotypes indicated that around 46% of DEGs were up-regulated and 57% DEGs were down regulated in CTV while 20% DEGs were up-regulated and 80% DEGs were down-regulated in CSV. Furthermore, comparison of CSV and CTV, DEGs involved in KEGG pathway indicates that around 49 DEGs were commonly involved in early response phase while 155 and 142 DEGs exclusively present in the contrasting two varieties revealed cold susceptible and cold tolerant DEGs, respectively. Furthermore, comparison at late response phase (T_4_) and recovery response phase (T_5_) revealed that 324 genes were up-regulated exclusively in T_4_ and 195 were down-regulated in T_5_ in which 4 genes (Os02g44230/Os02g44235, trehalose 6-phosphate phosphatase; Os07g42960, 3-deoxy-7-phosphoheptulonate synthase; Os08g06344, ATP-dependent RNA helicase DDX46/PRP5) were significantly down-regulated and up-regulated in T_5_ and T_4_, respectively (Fig. 3; Additional file 19: Table S13 and Additional file 20: Table S14). Interestingly, out of four genes, three genes (Os02g44230, Os02g44235, Os08g06344) were up-regulated in S_4_ (48h late response phase of susceptible genotype) and one gene (Os08g06344) was down-regulated in S_5_ (24 h recovery condition of susceptible genotype). More interestingly, out of 324 up-regulated genes of T_4_, 88 genes were also up-regulated in S_4_ genotype. In the susceptible genotype Sahabhagidhan, our results showed that 702 genes were significantly down-regulated in late response phase in which 11 genes (Os03g09020, S- hydroxymethyl glutathione dehydrogenase/alcohol dehydrogenase; Os04g38540, aldose 1-epimerase; Os12g03470, alpha-N-arabinofuranosidase; Os07g34520, isocitrate lyase; Os07g05365, photosystem II 10 kDa protein; Os12g08260, 2-oxoisovalerate dehydrogenase E1 component; Os05g43830, proline iminopeptidase; Os10g02070, peroxidase; Os03g15960, HSP20 family protein; Os04g53210, (S)-2-hydroxy-acid oxidase; Os03g22680, RING finger and CHY zinc finger domain-containing protein) were significantly up-regulated in tolerant genotype. Out of 11, 9 genes (except Os10g02070, Os03g15960) were commonly expressed in tolerant genotype at 24 h recovery phase. Furthermore, out of these two exclusive genes, one gene (Os03g15960) was also down-regulated in toerant genotype after 48 h late phase and another gene (Os10g02070) was exclusively down-regulated and up-regulated in CSV at 48 h late response phase and 24 h recovery phases, respectively (Additional file 4: Figure S4 and Additional file 5: Figure S5; Additional file 21: Table S15). These results indicated the fact that all 11 genes were significantly involved in different pathways and act as cold responsive genes.

Expression of genes (Os02g44230 and Os02g44235) encoding enzyme trehalose-6- phosphate phosphatase (TPP) were induced by cold response in CSV while reduced expression was observed in CTV at early response phase and 48 h late response phase. Down-regulation of these two genes was found after 24 h recovery condition of both in CSV and CTV genotypes. Genes (Os07g48160 and Os10g35110) encoding α-Gal were down-regulated at 48 h late response phase while up-regulated after 48 h recovery in CTV genotype. Further, gene encoding α-glucosidase (Os06g46284) and β-glucosidase (Os03g53800) were found down-regulated and up-regulated at 48 h late response phase and 24 h recovery in CTV genotype. In energy metabolism pathways, 24 and 11 DEGs were enriched in CTV and CSV at early response phase, respectively in which only 3 genes (Os04g56160, Os01g36720 and Os07g32570) were commonly down-regulated in both varieties. Furthermore, genes (Os04g16732, Os10g21394) encoding NAD (P) H-quiNone oxidoreductase subunit H (ndhH) were consistently up-regulated at each time interval of stress condition and down-regulated during 24 h recovery condition in CTV genotype. In addition, gene (Os07g42960) encoding 3-deoxy-7-phosphoheptulonate synthase, a chloroplast precursor involved in photosynthesis was significantly up-regulated in cold stress condition while down-regulated after 24 h recovery condition in CTV. Moreover, in our study, the rice gene (Os03g18070) encoding omega-3 fatty acid desaturase (desB) was significantly up-regulated during 48 h stress condition while down-regulated after 24 h recovery condition in CTV genotype.

### Role of transcription factors under cold stress

**Fig. 4:**
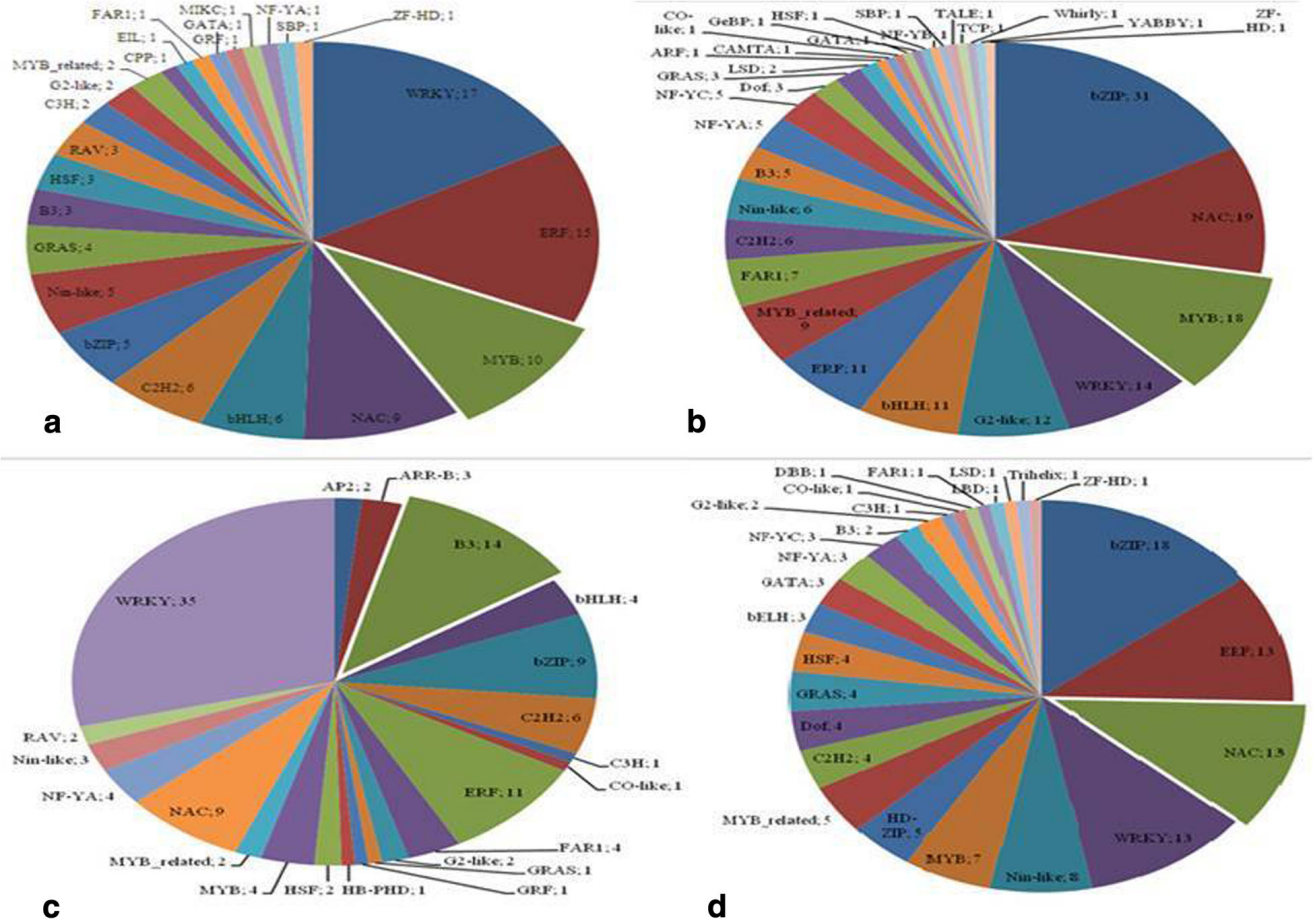
Differential expression of transcription factors genes involved during early response of CSV and CTV genotype. **a** up-regulated TF genes in CSV, **b** down-regulated TF genes in CSV, **c** up-regulated TF genes in CTV, **d** down-regulated TF genes in CTV

Of the 2438 known or annotated TF genes in rice genome [30], 1583 differentially expressed TF genes were identified in which 453, 704 and 444 were present in both genotypes at 6 h early response phase, 48 h late response phase and 24 h recovery condition, respectively (Fig. 4; Additional file 6: Figure S6; Additional file 22: Table S16). In early response phase, a total of 192, 154 and 25 TF genes were detected as CSV specific, CTV specific, cold responsive genes, respectively. During late response phase of under the stress, a total of 239 CSV specific; 231 CTV specific and 233 commonly regulated TF genes were detected. While during recovery phase, a total of 158 CSV specific; 189 CTV specific and 93 commonly regulated TF genes were detected after the stress condition. Among the transcription factors, 69 WRKY, 46 bZIP, 41 NAC, 40 ERF, 31/14 MYB/MYB-related, 22 bHLH, 17 Nin-like 7 HSF and 4C3H were involved during 6 h early response phase cold stress. In 48 h late response phase, TF genes include 30 bHLH, 65 NAC, 30 ERF, 26/20 MYB/MYB-related, 11 C3H, 12 HSF, 86 Nin-like, 41 AP2/ERF, 55 bZIP and 98 WRKY members (Additional file 23: Table S17). Furthermore, 18 bHLH, 50 NAC, 31 ERF, 24/13 MYB/MYB-related, 4 C3H, 4 HSF, 14 Nin-like, 31 bZIP and 114 WRKY TF genes involved after 24 h recovery. Interestingly, 4 TF genes (LOC_Os01g72680, LOC_Os04g10010, LOC_Os07g46920 and LOC_Os09g25070) were down-regulated in susceptible genotype while up-regulated in tolerant genotype at 6 h early response phase (Additional file 24: Table S18). Moreover, 1 TF gene (Os09g10054) was up-regulated in tolerant while down-regulated in susceptible genotype at 48 h late response phase (Additional file 24: Table S18). Besides, 4 TF genes (Os09g25070; Os03g03164; Os07g46920; Os11g02540) were up-regulated in tolerant while down-regulated in susceptible genotype after 24 h recovery condition (Additional file 25: Table S19).

The results revealed 41 differentially-regulated AP2/ERF genes at low temperature stress, amongst them 15 were commonly detected in both genotypes. There were 8 (Os01g04020; Os06g47590; Os06g10780; Os02g43820; Os02g45420; Os02g48184; Os02g45450; Os07g13170) and 7 (Os03g60430; Os10g42150; Os04g46400; Os10g22600; Os02g13710; Os06g11860; Os06g09688) AP2/ERF genes exclusively induced and repressed in CSV genotype by chilling stress, respectively while 11 (Os05g41780; Os04g48350; Os02g42585; Os04g44670; Os09g20350; Os10g41130; Os05g41760; Os02g43970; Os04g52090; Os02g51670; Os04g57340) genes were repressed in CTV genotype. Also, a set of WRKY genes were found to be chilling-regulated in both genotypes, of which 12 were commonly induced, 22 were exclusively up-regulated in CSV and 18 were exclusively up-regulated in CTV genotype. In the rice genome, there are many MYB-encoding genes with diverse roles in developmental processes and defense responses. In our study, we identified 46 MYB and MYB-related TF genes showing differential expression to low temperature stress in both the genotypes. Out of the total MYB genes detected, 29 were commonly regulated while 17 and 11 were exclusively regulated in CSV and CTV genotype, respectively. Further, we identified three MYB TF genes in which two genes (Os02g38980; Os04g49450) were induced and one gene (Os02g46030) repressed in CTV genotype under cold stress.

Further, TF genes in response to cold stress were analyzed from transcription factor database (STIFDB V2.0; http://caps.ncbs.res.in/stifdb2/) for early response, late response and recovery phases. Results showed that 6 ERF (Os01g21120; Os01g73770; Os02g43790; Os06g03670; Os08g36920; Os09g28440) and 1 MYB (Os02g41510), 3 C2H2 (Os03g41390; Os03g60560; Os03g60570) gens were up-regulated in CSV at 6 h early response phase. Moreover, 2 ERF (Os09g35010; Os09g35030), 2 MYB (Os03g20090; Os04g43680), 2 Nin-like (Os01g68490; Os10g35640), 2 WRKY (Os02g08440, Os05g25770), 1 C_2_H_2_ (Os03g32230), 1 NAC (Os07g34280) and 1 MYB-related (Os04g49450) TF genes were commonly up-regulated in CSV and CTV, revealing a positive regulatory role in response to low temperature stress in rice. Besides this, 2 TFs (ERF, Os03g64260; WRKY, Os02g32230) genes were up-regulated in CTV only. At 48 h late response phase, one TF gene (Os01g14870) C3H and two Nin-like TF genes (Os01g68490; Os10g35640) were induced while down-regulated WRKY (Os01g60640), CO-like (Os02g49870), HSF (Os08g43334) and NAC (Os11g05614) in response to cold stress in CTV. In addition, comparative analysis of CSV genotype showed TF gene C3H (Os01g14870), Nin-like (Os01g68490), C2H2 (Os03g32230; Os03g60560; Os03g60570), CO-like (Os08g08120) were down-regulated while CO-like (Os02g49870) and HSF (Os08g43334) were down-regulated. After 24 h recovery conditions in CTV genotype, TF NF-YA (Os06g46799), CO-like (Os08g08120), NAC (Os11g05614) were down-regulated in response to cold stress while WRKY (Os02g32230) was up-regulated.

### Quantitative real-time reverse transcription– PCR for validation of novel transcripts

**Fig. 5:**
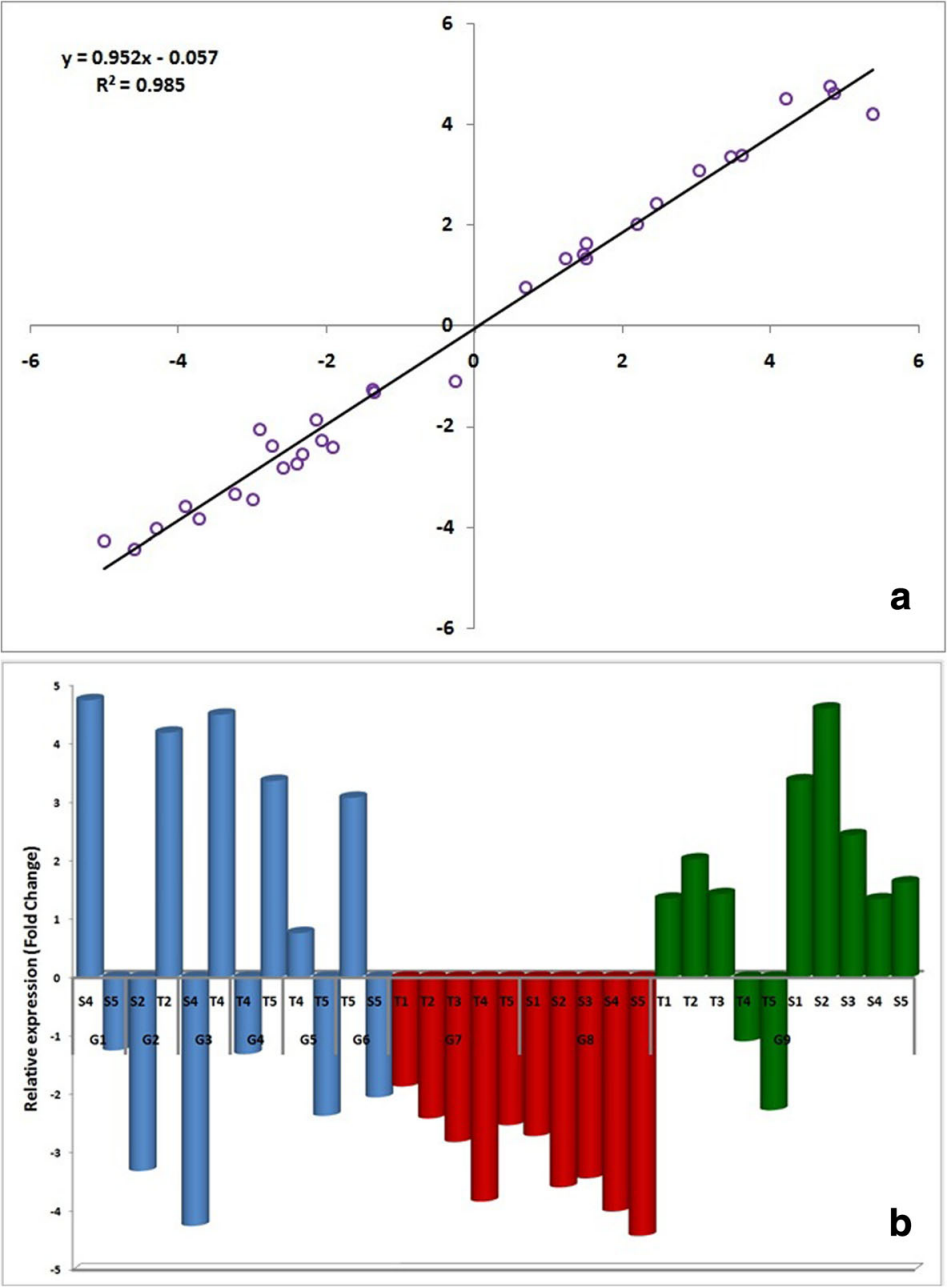
Validation of RNA-seq results through qRT-PCR analysis. **a** Correlation analysis of differentially expressed genes between qPCR analysis and RNA-seq experiment. **b** Fold change of differentially expressed genes through qPCR method (G1: LOC_Os02g44230.1, G2: LOC_Os09g25070.1, G3: LOC_Os09g10054.1, G4: LOC_Os03g53800.1, G5: LOC_Os03g18070.1, G6: LOC_Os03g03164.1, G7: LOC_Os05g42250.1, G8: LOC_Os02g33030.1, G9: LOC_Os02g08440.1)

The qRT-PCR analysis was performed for validating the results of RNA-seq sequencing as well as to test the differential expression of novel transcripts identified from the analysis. For the purpose the DEGs were selected carefully following certain criteria that (1) they must be highly up regulated or down regulated ones having at least 5-fold difference between the susceptible and tolerant genotype, (2) they should be differentially expressed being stage specific and/or genotype specific. On the basis of these criteria nine transcripts were considered for validation. The results of qRT-PCR for the genes LOC_Os02g44230.1, LOC_Os09g25070.1, LOC_Os09g10054.1, LOC_Os03g53800.1, LOC_Os03g18070.1, LOC_Os03g03164.1, LOC_Os05g42250.1, LOC_Os02g33030.1 and LOC_Os02g08440.1 normalized with housekeeping gene b-tubulin in response to different chilling treatments were validated. A positive correlation (R^2^ = 0.985) was observed between RNA-seq and qRT-PCR expression analyses (Fig. 5).

## Discussion

### Phenotyping for chilling stress tolerance

There are few reports for classifying rice genotypes based on seedling stage cold tolerance under low temperature regimes and duration [1, 32]. Various classes of genotypes to cold tolerance at the seedling stage were evaluated at 10 °C for 10 and 13 days [32]. Cold tolerance at seedling stage at 9 °C for 8, 14, 16 and 18 days were reported earlier [1]. However, rice genotypes classification with respect to seedling stage cold tolerance by exposing the seedlings to a temperature regime of 25 to 4 °C and further extending the exposure up to 15, 20 and 25 days at 4 °C were reported in previous study where the rice genotypes were grouped into six classes [2, 3]. Rice genotype, Geetanjali was categorized as very highly tolerant and Sahabhagidhan as highly susceptible type to chilling stress reported by earlier publications [2, 3]. Thus, both the studied genotypes for RNA-seq analyses were confirmed to be very highly contrasting for seedling stage chilling stress tolerance.

### RNA sequencing and DEGs in response to chilling stress

Previous study on seedling stage cold tolerance to identify underlying molecular genetics pathways imparting cold stress adaptation using microarray on affymetrix has been reported [25, 33]. The RNA-seq technology with an advantage of global representation and precise measurement of expression level of each gene in a sample by mapping short DNA sequences on a reference [34]. A total of 24529 DEGs in both the contrasting genotypes were observed (Fig. 1). Previous findings on cold and salinity tolerance also revealed relatively large number of DEGs in contrasting genotypes [34–36]. On the basis of CuumeRbund hierarchical cluster analysis of transcripts clearly divided the response to cold stress into three phases: an early response phase (6 h cold stress), late response phase (12, 24 and 48 h cold stress) and recovery phase (after 24 h). These results were consistent with the previous reports on cold stress gene profiling [25, 33, 34]. Further, comparative analysis of DEGs of CSV and CTV revealed 219 and 116 DEGs commonly involved at each time interval, respectively in which 198 and 95 DEGs were exclusively present in CSV and CTV genotypes and 21 DEGs were commonly involved in both varieties (Fig. 1). When we compared the expression profile of DEGs of both the genotypes, significant numbers of DEGs were down-regulated as compared to up-regulated genes except 48 h recovery condition of CTV (Additional file 2: Figure S2; Additional file 9: Table S3).

Comparative gene expression level on the basis of FC revealed 24.97 and 75.03% genes up-regulated and down-regulated in CSV while 43.92 and 56.08% genes were up-regulated and down-regulated in CTV, respectively (Additional file 9: Table S3; Additional file 10: Table S4). This signifies that CTV harbors potential to specifically maintain the higher percentage of genes up-regulated during the course of cold stress. This suggests that presence of these genes may confer tolerance to CTV’s during exposure to cold stress. Similar result was observed for K354 and C418, during cold stress, the favorable alleles from *indica* donor Bg300 improved the cold tolerance of K354 over C418 [25]. Our results also showed that more number of genes participated to recover CTV from cold stress. Further, in CTV, both increase and decrease in exclusively expressed genes were observed during various response phases, but in 48 h recovery condition a sharp decline was observed, indicating that plant regained the expression pattern similar to control condition during this short recovery period, while still maximum number of genes were involved in CSV during this phase also, indicating large number of genes were involved in its survival (Fig. 3; Additional file 2: Figure S2). For normal growth and development, 2207 and 4858 genes were exclusively expressed in CSV and CTV (Fig. 1). Thus, it may be concluded that more number of genes were involved in becoming the genotype as tolerant. Earlier studies on gene expression have suggested that the highly constitutive gene expression before exposure to abiotic stress might represent a constitutive tolerance in tolerant genotypes [37–40]. Many research findings have suggested that the major group comprises genes unresponsive to chilling stress in either genotype as like 154 genes that were heavily detected in CTV, such as those encoding glutathione stransferase, oxidoreductase and thioredoxin, those increased chilling tolerance by maintaining cell redox homeostasis [41–44].

### Functional characterization of DEGs

In molecular function category, 3 GO common terms were enriched in both the genotypes. This indicated few common molecular functions in both CSV and CTV. Under chilling stress, the susceptible genotype ‘Sahabhagidhan’ was not enriched with catalytic activity, hydrolase activity and lipid binding molecular function. Hence, under stress, genotype that lacks these functions may be susceptible to the stress. The cellular component characterization showed six GO terms enriched of which 3 GO terms were significantly involved in all three phase of CSV while in CTV, these were enriched in 24 h recovery condition. This indicates the absence of photosynthesis related genes, storage products like starch and synthesis of other classes of molecules like fatty acids and terpenes used for energy production and as raw material for the synthesis of other molecules during cold stress condition in CSV. In biological process category, lipid metabolic process showed the same biological function in both susceptible and tolerant genotypes while signal transduction, regulation of cellular process, pollen-pistil interaction, regulation of biological process, photosynthesis, generation of precursor metabolites and energy, carbohydrate metabolic process were detected and enriched in early response phase of CTV. This indicates that significant numbers of DEGs are involved in genotype as tolerant during cold stress (Table 1; Additional file 12: Table S6).

**Table 1:** Significant GO terms enriched DEGs of cold tolerant variety (CTV) on the basis of FDR corrected *p*-value of three different functional categories

GO term	Category	Description	*p-value*		
			Early response	Late response	24 h recovery
GO:0050896	Biological process	Response to stimulus	1.50E-012	4.10E-006	4.30E-011
GO:0009719	Biological process	Response to endogenous stimulus	5.50E-011	6.30E-005	7.00E-005
GO:0006950	Biological process	Response to stress	4.30E-010	2.60E-005	7.70E-009
GO:0019748	Biological process	Secondary metabolic process	4.30E-008	NE^*^	9.20E-005
GO:0009607	Biological process	Response to biotic stimulus	3.10E-007	4.80E-005	6.10E-007
GO:0009628	Biological process	Response to abiotic stimulus	3.20E-006	0.00016	2.20E-007
GO:0007165	Biological process	Signal transduction	0.0002	NE^*^	NE^*^
GO:0050794	Biological process	Regulation of cellular process	0.0002	NE^*^	NE^*^
GO:0065007	Biological process	Biological regulation	0.00034	NE^*^	NE^*^
GO:0009875	Biological process	Pollen-pistil interaction	0.00086	NE^*^	NE^*^
GO:0050789	Biological process	Regulation of biological process	0.0035	NE^*^	NE^*^
GO:0015979	Biological process	Photosynthesis	NE^*^	3.3E-015	NE^*^
GO:0006091	Biological process	Generation of precursor metabolites and energy	NE^*^	1.70E-007	NE^*^
GO:0006629	Biological process	Lipid metabolic process	NE^*^	NE^*^	0.00044
GO:0005975	Biological process	Carbohydrate metabolic process	NE^*^	NE^*^	0.00047
GO:0019825	Molecular function	Oxygen binding	2.10E-007	9.60E-006	8.90E-011
GO:0030246	Molecular function	Carbohydrate binding	2.60E-005	NE^*^	NE^*^
GO:0016740	Molecular function	Transferase activity	0.00026	NE^*^	NE^*^
GO:0003700	Molecular function	Transcription factor activity	0.0003	0.0012	3.00E-005
GO:0016301	Molecular function	Kinase activity	0.00083	NE^*^	NE^*^
GO:0016772	Molecular function	Transferase activity, transferring phosphorus-containing groups	0.00083	NE^*^	NE^*^
GO:0003824	Molecular function	Catalytic activity	0.006	NE^*^	0.00017
GO:0005215	Molecular function	Transporter activity	NE^*^	NE^*^	0.0035
GO:0016787	Molecular function	Hydrolase activity	NE^*^	NE^*^	0.00082
GO:0009579	Cellular component	Thylakoid	NE^*^	3.60E-012	NE^*^
GO:0030312	Cellular component	External encapsulating structure	NE^*^	NE^*^	0.0018
GO:0005618	Cellular component	Cell wall	NE^*^	NE^*^	0.0014
GO:0005576	Cellular component	Extracellular region	NE^*^	NE^*^	0.0037

NE^*^ denotes significantly not enriched genes

In early response phase, a very high number of DEGs were enriched in biological and molecular process in CTV as compared to CSV. Therefore, Getanjali showed tolerance response to early phase cold stress. However, no DEGs were enriched in CTV for cellular component, while 272 DEGs were enriched in 3 categories. This indicated that in CTV, cold stress though changed the expression profiles of genes involved in molecular and biological process, the genes related to cellular component remained unchanged indicating inherent strong morphological features to with stand cold stress. In both CSV and CTV, 798 and 688 DEGs were represented for response to stress and stimulus, respectively. The DEGs effects were different in both the genotypes; in CTV it induced many DEGs under several categories except for cellular components, on contrary in CSV, no DEGs were represented for many of the important biological components: metabolic processes and signal transduction; molecular functions: catalytic activity, kinase activity, and protein binding. In cellular component, 102 DEGs of CSV were represented mainly for cell wall and all of them were down regulated (Fig. 2; Table 2; Additional file 3: Figure S3; Additional file 13: Table S7; Additional file 16: Table S10). During 12-48 h of cold stress (late response phase), similar to early response DEGs, more DEGs were enriched in response to stress and stimulus. In this response phase, cellular component showed enrichment in susceptible genotype. While, tolerant genotype was enriched in photosynthesis, generation of precursor metabolites and energy, thylakoid, transporter activity, transcription regulator activity, oxygen binding and transcription factor activity, along with new DEGs representing in photosynthesis, generation of precursor metabolites and energy, catalytic activity, kinase activity, biological regulation, signal transduction, transferase activity-transferring phosphorus-containing groups, regulation of cellular process, secondary metabolic process, regulation of biological process, lipid metabolic process, carbohydrate binding, and pollen-pistil interaction got stabilized during the late cold stress (Fig. 2; Additional file 14: Table S8; Additional file 17: Table S11). Thus, genotype Getanjali remain tolerant in response to chilling stress. Previous research finding reveals that DEGs are genotype specific and observed to be regulated in the late phase of chilling stress treatment [25]. In recovery phase, the tolerant genotype showed more functional category enrichment than susceptible genotype particularly in response to stimulus and stress, metabolic process, lipid and carbohydrate metabolism and transcription factor/regulator activity, whereas in CSV, DEGs related to lipid binding, thylakoid, and plastid were exclusive (Additional file 15: Table S9; Additional file 18: Table S12). Hence, the genotype Getanjali is tolerant to chilling stress. Previous report revealed that commonly up-regulated genes during recovery conditions enriched in metabolic, oxidation-reduction, and stress-response processes, indicates their general role in recovery mechanisms of rice plants after chilling stress [25].

**Table 2:** Comparison of up-regulated and down-regulated genes of CSV and CTV at different time interval

Treatment time	Sample	Up-regulation	Down-regulation
6 h	S1_exclusive	243	1335
	T1_exclusive	586	474
	S1_T1_both	181	414
12 h	S2_exclusive	261	2089
	T2_exclusive	371	465
	S2_T2_both	223	695
24 h	S3_exclusive	397	1887
	T3_exclusive	529	365
	S3_T3_both	372	559
48 h	S4_exclusive	555	1360
	T4_exclusive	550	916
	S4_T4_both	533	1160
24 h recovery	S5_exclusive	284	646
	T5_exclusive	881	589
	S5_T5_both	429	307

### KEGG pathway annotation

It was found that cold treatment continuously increased the number of up-regulated and down-regulated genes in both CSV and CTV genotypes, while it decreased after 24 h recovery, except exclusively expressed genes of CTV during recovery (Table 2). These results indicate that prolonged cold stress (6-48 h) increased number of up-regulated and down-regulated genes that might be involved in tolerance/susceptibility response. Further, decrease in number of up-regulated and down-regulated genes after 24 h recovery indicated that plant adapted to normal environment and suppressed expression of stress related genes. Therefore, we analyzed early response phase, late response phase specifically at 48 h cold stress and 24 h recovery to reveal the role of genes during cold stress and after recovery, normalized with control condition in both the genotypes. Comparative analysis of two genotypes revealed 29216 and 30867 genes expressed in susceptible and tolerant genotypes, respectively under control condition. These results indicate that the CTV contain higher DEGs in different pathways by which the genotype tolerate better under the stress condition. The commonly expressed genes (26009) in both varieties may be involved in plant growth and development. Comparison of early response phase in both varieties indicated that around 46% of DEGs were up-regulated and 57% DEGs were down regulated in CTV while 20% DEGs were up-regulated and 80% DEGs were down-regulated in CSV. These results indicate that tolerant genotype contains high percentage of up-regulated genes than susceptible genotype making the genotype tolerant.

Earlier research findings have suggested that cold responsive genes encode proteins involved in enzymes for respiration and metabolism of carbohydrates, lipids, phenylpropanoids and antioxidants, molecular chaperones and antifreeze proteins [45, 46]. Trehalose acts against stress protection of metabolite and for storage of carbohydrate. In plants, trehalose biosynthesis is catalyzed by trehalose-6-phosphate synthase (TPS) and trehalose- 6- phosphate phosphatase (TPP). Earlier finding suggests that TPP genes express in rice and their expression is influenced by cold stress [47]. Chilling stress accumulates trehalose rapidly and transiently due to transient induction of TPP activity in rice tissues. Over expression of TPS and TPP genes increased the level of trehalose accumulation and showed more tolerance to cold stress in transgenic tobacco and rice [48–51]. Our results showed that expression of genes (Os02g44230 and Os02g44235) encoding enzyme TPP were induced by cold response in CSV while reduced expression was observed in CTV at early response phase and 48 h late response phase. Down-regulation of these two genes were found after 24 h recovery condition of both CSV and CTV, suggesting the fact that cold tolerant plant synthesize increased amount of TPP/TPS for regulating cell shape and plant architecture. In addition, genes (Os02g51680 and Os09g20390) encoding enzyme TPP/TPS were down-regulated in CSV. In transgenic petunia, down-regulation of α-Gal (α-Galactosidase) exhibited more tolerance under chilling stress suggested for engineering of raffinose metabolism through transformation with α–Gal [52]. Our results showed that genes (Os07g48160 and Os10g35110) encoding α-Gal were down-regulated at 48 h late response phase while up-regulated after 48 h recovery in CTV. Further, gene encoding α-glucosidase (Os06g46284) and β-glucosidase (Os03g53800) were found down-regulated and up-regulated at 48 h late response phase and 24 h recovery in CTV.

In energy metabolism pathways, 24 and 11 DEGs were enriched in CTV and CSV at early response phase, respectively in which only 3 genes (Os04g56160, Os01g36720 and Os07g32570) were commonly down-regulated in both varieties. Results indicated that a large proportion of genes were exclusively expressed and involved in different pathways of CTV, which were not participated in the pathway of CSV and made a substantial difference between tolerant and susceptible types in early response phase. Furthermore, genes (Os04g16732, Os10g21394) encoding NAD (P) H-quiNone oxidoreductase subunit H (ndhH) were consistently up-regulated at each time interval of stress condition and down-regulated during 24 h recovery condition in CTV. These results indicated that NDH-dependent cyclic pathway around PSI participates in ATP supply in conditions of high ATP demand in response to severe stress conditions. In addition, gene (Os07g42960) encoding 3-deoxy-7-phosphoheptulonate synthase, a chloroplast precursor involved in photosynthesis was significantly up-regulated in cold stress condition while down-regulated after 24 h recovery condition in CTV. Moreover, in our study, the rice gene (Os03g18070) encoding omega-3 fatty acid desaturase (desB) was significantly up-regulated during 48 h stress condition while down-regulated after 24 h recovery condition in CTV indicating the fact that increased linolenic acid levels are essential for the maintenance of membrane fluidity and chloroplast function under chilling stress exposure [53]. Cold stress also leads to change in the expression profile of enzyme peroxidase (POD) and their activity increases under low temperature [54]. Our results showed that gene encoding POD (Os01g15830) was significantly down-regulated at 6 h, 24 h, 48 h and 24 h recovery conditions in CSV while up-regulated at 24 h and after 24 h recovery conditions in CTV. In regard to catalase (CAT) activity, gene expression profile of both varieties was observed differently. Gene encoding catalase (Os06g51150 and Os02g02400) was significantly up-regulated after 24 h recovery condition in CTV while down-regulated during 48 h stress condition in CSV. Previous results also suggested different expression profile of gene encoding CAT in chilling stress microarray study of rice [25].

We identified gene, Os08g06344 encoding ATP-dependent RNA helicase, that acts on acid anhydrides to facilitate cellular and sub cellular movement, was up-regulated at 6 h and 48 h late response phase in CTV and may be responsible for cold tolerance in rice. Low expression of osmotically responsive gene (LOS4), one of the helicase encoded by the Arabidopsis, is responsible for tolerance to chilling and freezing stress [55]. Defects in the nucleocytoplasmic transport of RNA seem to affect cold tolerance preferentially, because the LOS4 mutant plants do not have severe growth or developmental phenotypes, nor they are strongly altered in other abiotic stresses. Further, we indentified 12 differentially expressed genotype specific genes in which 4 DEGs (Os08g44360; Os02g54140; Os02g07110; Os05g42250) were exclusively present in CTV and 8 DEGs (Os05g03610; Os02g33030; Os10g34480; Os01g39860; Os09g04050; Os02g09490; Os06g14240; Os05g03610) were exclusively present in CSV. The roles of these genes need to be further examined.

### Role of transcription factors under cold stress

A large group of transcription factors are involved in response to abiotic stresses comprising the AP2/EREBP family [31, 56]. In our study, we detected 41 differentially-regulated AP2/ERF genes at low temperature stress, amongst them 15 were commonly detected in both varieties. These results are in conformity with the previous reports on low temperature stress regulation due to presence of CBF/DREB subfamily [57–59]. More than half of the 103 WRKY TF genes have been identified in the rice varieties that are shown to be transcriptionally regulated under conditions of biotic and/or abiotic stresses [60, 61]. In our study, a set of WRKY genes were found to be chilling-regulated in both genotypes, of which 12 were commonly induced, 22 were exclusively up-regulated in CSV and 18 were exclusively up-regulated in CTV. Three chilling-induced WRKY genes (Os05g0343400, Os01g0246700, and Os01g0826400) in both varieties were also observed to be induced by chilling stress in a *japonica* rice genotype [57]. The remaining WRKY TF genes were differentially regulated involved in differential response of the varieties.

In our study, we identified 46 MYB and MYB-related TF genes showing differential expression to low temperature stress in both the varieties. Out of the total MYB genes detected, 29 were commonly regulated while 17 and 11 were exclusively regulated in CSV and CTV, respectively. Eleven of these MYB/MYB-related genes have been reported in *japonica* rice in response to chilling stress [57]. There are many evidences in arabidopsis and rice to support for the involvement of many MYB proteins in response to abiotic stress particularly to seedling stage cold stress [21, 62–64]. Therefore, our detected MYB TF genes are functionally involved in response to chilling stress. We found that OsMYB4 (Os04g43680) was induced in response to chilling stress in CSV. Earlier finding also suggest that Myb4 over expressing plants show increased cold tolerance [62]. Further, we identified three MYB TF genes in which two genes (Os02g38980; Os04g49450) were induced and one gene (Os02g46030) repressed in CTV under cold stress.

Further, TF genes in response to cold stress were analyzed from transcription factor database (STIFDB V2.0; http://caps.ncbs.res.in/stifdb2/) for early response, late response and recovery phases. Our results showed that 6 ERF and 1 MYB, 3 C2H2 gens were up-regulated in CSV at 6 h early response phase. Moreover, 2 ERF, 2 MYB, 2 Nin-like, 2 WRKY, 1 C_2_H_2_, 1 NAC and 1 MYB-related TF genes were commonly up-regulated in CSV and CTV, revealing a positive regulatory roles in response to low temperature stress in rice. Besides this, ERF (Os03g64260) and WRKY (Os02g32230) genes were up-regulated in CTV only. Earlier report indicated that TFs (AP2/EREBP, MYB, HSF, and NAC) regulated most of the genes after 2 h chilling stress [25]. At 48 h late response phase, one TF gene (Os01g14870) C3H and two Nin-like TF genes (Os01g68490; Os10g35640) were induced while down-regulated WRKY (Os01g60640), CO-like (Os02g49870), HSF (Os08g43334) and NAC (Os11g05614) in response to cold stress in CTV. In addition, comparative analysis of CSV showed TF gene C3H (Os01g14870), Nin-like (Os01g68490), C2H2 (Os03g32230; Os03g60560; Os03g60570), CO-like (Os08g08120) were down-regulated while CO-like (Os02g49870) and HSF (Os08g43334) were down-regulated. After 24 h recovery conditions in CTV, TF NF-YA (Os06g46799), CO-like (Os08g08120), NAC (Os11g05614) were down-regulated in response to cold stress while WRKY (Os02g32230) was up-regulated. In CSV, C2H2 (Os03g60560; Os03g60570) was up-regulated while MYB-related (Os02g46030) and NF-YA (Os06g46799) were down-regulated [65–67].

### Quantitative real-time reverse transcription– PCR for validation of novel transcripts

For validating the results of RNA-seq sequencing, qRT-PCR was performed to test the differential expression of novel transcripts identified from analysis. The results of qRT-PCR for the nine genes normalized with housekeeping gene b-tubulin in response to different chilling treatments were validated. A positive correlation (R^2^ = 0.985) was observed between RNA-seq and qRT-PCR expression analyses (Fig. 5). This confirmed the reliability of expression of differentially expressed genes identified through RNA-seq.

## Conclusions

The down regulated genes were more in susceptible genotype in all 3 phases while up regulated genes were more in tolerant genotype. The down-regulated genes declined during recovery in CSV and CTV. The numbers of up-regulated genes were more in CTV during recovery phase under cold stress. A significant involvement of transcription regulation, oxygen and lipid binding, catalytic and hydrolase activity was established in gene ontology classification. Absence of photosynthesis related genes, storage products like starch and synthesis of other classes of molecules like fatty acids and terpenes used for energy production and as raw material for the synthesis of other molecules during cold stress condition were observed in cold susceptible varieties. During recovery phase of cold stress, no genes for photosynthesis and storage products like starch and the synthesis of many classes of molecules are detected in the CSV. Under biological process category, response to biotic stimulus, response to abiotic stimulus, secondary metabolic process and lipid metabolic process were influenced for both the varieties. Catalytic activity, hydrolase activity and lipid binding molecular function are absent in susceptible genotype. More number of DEGs were involved in different pathways of tolerant genotype imparting the genotype as tolerant to chilling stress. A total of 41 differentially-regulated AP2/ERF transcription factors genes were detected at low temperature stress. A set of WRKY genes comprising 12 were commonly induced, 22 were exclusively up-regulated in CSV and 18 were exclusively up-regulated in CTV. Also, 46 MYB and MYB-related TF genes were identified showing differential expression to low temperature stress in both the varieties.

## Methods

### Plant materials and growth conditions

The highly tolerant genotype Geetanjali and susceptible cultivar, Sahabhagidhan to vegetative stage cold tolerance were collected from ICAR-National Rice Research Institute, Cuttack, Odisha, India and raised in pots till 21 days. The potted plants were exposed to cold stress condition in a walk-in cold growth chamber (Conviron PGV36). In the growth chamber, 75–85% relative humidity (RH), 800 μ moles s^− 1^ m^− 2^ light intensity above 60 cm from the floor and 12 h photoperiod were maintained. Phenotyping for seedling stage cold tolerance in the contrasting varieties was performed as per the published protocol [2, 3]. In the protocol, seedlings were grown in RGA-cum-Phytotron till three-leaf stage at a set temperature of 25 °C and photoperiod of 12 h. During this stage, the weak seedlings were removed and healthy seedlings were exposed to low temperature regime (LTR) treatments in the growth chamber with the pre setting of temperature and RH as in the protocol. The low temperature regime1 (LTR1) was applied by exposing the seedling to a temperature of 25 °C exposed for 3 days; low temperature regime 2 (LTR2) was achieved by a gradual decrease in temperature to 15 °C within 7 days from 25 °C and maintained at 15 °C; low temperature regime 3 (LTR3) exposed to gradual decrease in temperature to 8 °C from 15 °C within 7 days and maintained at 8 °C; low temperature regime 4 (LTR4) with a gradual decrease in temperature to 4 °C from 8 °C within 7 days and maintained at 4 °C; low temperature regime 5 (LTR5) exposed to temperature of 4 °C for 14 days and low temperature regime 6 (LTR6) to temperature of 4 °C for 21 days. The varieties were evaluated using a modified IRRI-SES score under control facility.

Leaf samples of both the varieties were collected at 25 °C, after 6 h, 12 h, 24 h, 48 h at 4 °C exposure and recovery was observed after 48 h at 4 °C and kept for 24 h at 25 °C. The leaf samples from three different biological replicates were collected in different vials containing RNA stabilizer solution (Xcelris, India) and immediately stored at − 80 °C. 1–2 leaves from each seedling were collected in order to include maximum number of plants in single biological replicate. The three biological replicates included three separate set of chilling stress treatment experiments. For total mRNA isolation 100 mg of tissue from each biological replicates were taken together and crushed with liquid nitrogen to make powder form. 100 mg of the this powdered tissue was taken for further mRNA isolation followed by RNAseq analysis. The treatments for RNA-seq analyses were S_0_ to S_5_ and T_0_ to T_5_ for CSV and CTV at 0, 6, 12, 24, 48 and 24 h recovery after 48 h cold stress conditions, respectively. Zero hour stress indicates normal/control condition (S_0_/T_0_) whereas S_1_ to S_5_ and T_1_ to T_5_ represent the treatments.

### RNA isolation and sequencing

The leaf samples from three different biological replicates were collected in different vials containing RNA stabilizing solution. 1–2 leaves from each seedling were collected in order to include maximum number of plants in single biological replicate. The three biological replicates included three separate set of chilling stress treatment experiments. For mRNA isolation 100 mg of tissue from each biological replicates were taken together and crushed with liquid nitrogen to make powder form. 100 mg of the this powdered tissue was taken for further RNA isolation followed by RNAseq analysis. Total mRNA was isolated XcelGen total RNA isolation kit (Xcelris Genomics, India) as per manufacturer’s instructions. The yield and purity of RNA were evaluated by recording absorbance at 260 and 280 nm on Nanodrop 8000 Spectrophotometer (Thermoscientific). Further, the integrity of RNA was checked using RNA 6000 Nano LabChip using Agilent Bioanalyzer 2100 (Agilent Technologies, Germany). The pair end sequencing libraries for RNA-seq were prepared using illumina TruSeq® RNA Library Preparation Kit as per manufacturer’s protocol (illumina®, San Diego, CA). Library quality control and quantification were performed on Caliper LabChip GX using HT DNA High Sensitivity Assay Kit. RNA-Seq was performed using illumina HiSeq2000 (illumina®, San Diego, CA) as per manufacturer’s protocol to generate 30 million 2 × 100 bp reads for each sample. The processing of fluorescent images into sequences, base-calling and quality value calculations were performed using the illumina data processing pipeline (version 1.8).

### Reads mapping and annotations

Rice genome and gene information for reference cultivar, Nipponbare (*Oryza sativa L. subsp*. *japonica*) was downloaded from ftp:// ftp.plantbiology.msu.edu/pub/data/EukaryoticProjects/osativa/annotationdbs/pseudomolecules/version_7.0/all.dir/. High quality (QV > 25) transcriptome libraries from all 12 samples were individually mapped to the *rice* genome using TopHat v1.3.3 and Bowtie v0.12.9 with default parameters. Transcript abundance and differential gene expression were estimated using the programme Cufflinks v1.3.0 alongwith FPKM (Fragments Per Kilobase of transcript per Million mapped reads) from reference-guided mapping [68]. Genes expressed at very low levels (read counts < 10 across all 12 libraries) were excluded from differential gene expression analysis. Significance tests for differential expression were based on a modified exact test. Differentially expressed genes were identified at 0.05 false discovery rate (FDR) 0.05 and *p*-value ≤0.05. The CuumeRbund, a package of R was used to produce the heat maps of differentially expressed genes [69].

### Gene ontology (GO), KEGG Orthology (KO), and enrichment analysis

Functional classification of the DEGs was performed using *agriGo;* GO analysis toolkit and database using singular enrichment analysis (SEA) against supported species *Oryza sativa* MSU build 7.0 and further annotations were plotted using Web Gene Ontology Annotation Plot (WEGO) software. Statistical methods like Hypergeometric was used to obtain significant GO-terms considering *P*-value < 0.05. Hypergeometric tests with Benjamini and Hochberg FDRs were used for analysis taking the default parameters to adjust the P-value. Functional annotation of differentially expressed genes was done by BLAST comparisons against KEGG GENES database KAAS (KEGG Automatic Annotation Server). The KO terms were assigned (representative gene data set for rice *Oryza sativa* Ref.Seq) by using the SBH (Single-directional best hit) option. Pathway mapping was done using the KEGG Orthology database (http://www.genome. jp/kegg/ko.html) [70].

### Transcription factor identification

For the identification of transcription factor families represented in rice transcriptome, 10639 DEG were searched against 2438 transcription factors of *Oryza sativa subsp. japonica*, classified into 56 families at rice transcription factor database (PlantTFDB v2.0) using BLASTX with an E-value cut-off of 1E-05.

### Quantitative real-time reverse transcription–PCR for validation of novel transcripts

The expression analysis for validation of novel transcripts involved in chilling stress, the transcript analysis for genes LOC_Os02g44230.1, LOC_Os09g25070.1, LOC_Os09g10054.1, LOC_Os03g53800.1, LOC_Os03g18070.1, LOC_Os03g03164.1, LOC_Os05g42250.1, LOC_Os02g33030.1 and LOC_Os02g08440.1 performed with real-time method. Twenty one days old rice seedlings were exposed to temperature at 25 °C, after 6 h, 12 h, 24 h, 48 h at 4 °C exposure and recovery after 48 h at 4 °C and kept for 24 h at 25 °C. Total mRNA was isolated from the stressed and non-stressed plants with biological replicates using Trizol LS reagent (Qiagen) as per the manufacturer’s instructions. cDNA was synthesized by using oligo-dT primers with SuperscriptII (Invitrogen) cDNA synthesis kit. Expression analysis of Os02g44230.1, Os09g25070.1, Os09g10054.1, Os03g53800.1, Os03g18070.1, Os03g03164.1, Os05g42250.1, Os02g33030.1 and Os02g08440.1 in response to duration of stress was performed by real-time analysis with real-time primers (Additional file 26: Table S20). β-tubulin gene from rice was taken for normalization. Fold change in expression of the genes in various hours of cold stress conditions as compared to controlled condition were calculated by using 2^-ΔΔ Ct^ method [71]. Final expression result was obtained from three independent biological replicates and three technical replicates.

## Additional files

Additional file 1: **Figure S1.** Hierarchical cluster analysis of transcripts in CSV and CTV. The median ratio (stressed/control) was log (base 2)-transformed and subjected to linkage hierarchical clustering. S1-S5 and T1-T5 denote CSV and CTV at 6, 12, 24, 48 and 24 h recovery after 48 h stress conditions, respectively. (TIF 724 kb)

Additional file 2: **Figure S2.** Expression profiles of significant DEGs (up-regulated, down-regulated) and exclusively expressed genes in both genotypes i.e. CSV and CTV. Up and down-regulated genes were identified by comparing the fold change using FPKM values with control condition (S0). T denotes tolerant variety, Geetanjali (CTV) and S denotes susceptible variety, Sahabhagidhan (CSV). (JPG 44 kb)

Additional file 3: **Figure S3.** GO analysis of DEGs in early, late and 24 h recovery stages of susceptible cultivar, ‘Sahabhagidhan’. (JPG 62 kb)

Additional file 4: **Figure S4.** Venn diagram of DEGs under 6 h, 48 h and after 24 h recovery. A) Down-regulated genes of S1 and T1 B) Up-regulated genes of S1 and T1 C) Down-regulated genes of S4 and S5, D) Up-regulated genes of S4 and S5, E) Commonly down-regulated expressed genes of S4-T4 and S5-T5, F) Commonly up-regulated expressed genes of S4-T4 and S5-T5, G) Down-regulated genes of T4 and T5, H) Up-regulated genes of T4 and T5. S, T, 1, 4 and 5 denote CSV, CTV, 6 h, 48 h stress condition and 24 h recovery, respectively. (JPG 33 kb)

Additional file 5: **Figure S5.** KEGG pathway analysis of DEGs of CSV genotype at 6 h early response phase (S1), 48 h late response phase (S4) and 24 h recovery (S5). (JPG 61 kb)

Additional file 6: **Figure S6.** Differential expression of transcription factors genes involved during 48 h late response cold stress and 24 h recovery conditions of CSV genotype. a) up-regulated TF genes in S4, b) down-regulated TF genes in S4, c) up-regulated TF genes in S5, d) down-regulated TF genes in S5. (JPG 82 kb)

Additional file 7: **Table S1.** Summary of RNA-Seq reads and their mapping on the rice genome. (DOCX 22 kb)

Additional file 8: **Table S2.** Differentially expressed genes (DEGs) of CSV and CTV genotype at each time interval. (DOCX 13 kb)

Additional file 9: **Table S3.** Comparison of DEGs of CSV and CTV genotypes at each time interval. (DOCX 14 kb)

Additional file 10: **Table S4.** Differentially expressed genes (DEGs) of S1, S2, S3, S4 and S5 cold susceptible cultivar (CSV) against control sample (S0). (XLS 4297 kb)

Additional file 11: **Table S5.** Differentially expressed genes (DEGs) of T1, T2, T3, T4 and T5 cold tolerant cultivar (CTV) against control sample (T0). (XLS 3788 kb)

Additional file 12: **Table S6.** Top DEGs detected at different response phases of chilling stress in both the constrasting parents. (XLS 834 kb)

Additional file 13: **Table S7.** Significant GO terms enriched DEGs of cold susceptible variety (CSV) on the basis of FDR corrected *p*-value of three different functional categories. (DOCX 23 kb)

Additional file 14: **Table S8.** Significant GO terms of early response phase (S1) of CSV genotype. (DOCX 17 kb)

Additional file 15: **Table S9.** Significant GO terms of late response phase (S2-S4) of CSV genotype. (DOCX 13 kb)

Additional file 16: **Table S10.** Significant GO terms of 24 h recovery condition (S5) of CSV genotype. (DOCX 13 kb)

Additional file 17: **Table S11.** Significant GO terms of early response phase (T1) of CTV genotype. (DOCX 14 kb)

Additional file 18: **Table S12.** Significant GO terms of late response phase (T2-T4) of CTV genotype. (DOCX 13 kb)

Additional file 19: **Table S13.** Significant GO terms of 24 h recovery condition (T5) of CTV genotype. (DOCX 13 kb)

Additional file 20: **Table S14.** KEGG Pathway analysis of DEGs at 48 h late response phase and after 24 h recovery of CTV and their comparison with CSV genotype. (XLS 528 kb)

Additional file 21: **Table S15.** KEGG Pathway analysis of DEGs at 48 h late response phase and after 24 h recovery of CSV and their comparison with CTV genotype. (XLS 489 kb)

Additional file 22: **Table S16.** Distribution of differentially expressed transcription factors genes in 48 h late response phase and 24 h recovery condition in CSV and CTV genotypes. (XLS 35 kb)

Additional file 23: **Table S17.** Differentially expressed TF involved at 48 h late response phase and 24 h recovery conditions of both CSV and CTV genotypes. (XLS 32 kb)

Additional file 24: **Table S18.** Differentially expressed TF involved at 48 h late response phase of both CSV and CTV genotypes with annotation. (XLS 88 kb)

Additional file 25: **Table S19.** Differentially expressed TF involved in after 24 h recovery condition of both CSV and CTV genotypes with annotation. (XLS 66 kb)

Additional file 26: **Table S20.** List of primers used for qRT-PCR study. (XLSX 12 kb)

## Data Availability

Extra data has been appended as supplementary Tables. The accession number for sequence data generated in this study is PRJNA288892 available at http://trace.ncbi.nlm.nih.gov/.

## Abbreviations

AP2: Apetala 2
AREB: ABA responsive element
CBF: Cold stress binding factors
CSV: Cold susceptible variety
CTV: Cold tolerant variety
DEG: Differentially expressed genes
DREB: Dehydration responsive element binding proteins
ERF: Ethylene responsive element
FC: Fold change
FDR: False discovery rate
FPKM: Fragments per kilo base of transcript per million mapped reads
GO: Gene ontology
HSF: Heat shock factor
KEGG: Kyoto encyclopedia of genes and genomes
KO: KEGG Orthology
LTR: Low temperature regime
NAD: Nicotinamide adenine dinucleotide
qRT-PCR: Quantitative real time reverse transcription polymerase chain reaction
RNA: Ribonucleic acid
ROS: Reactive oxygen species
SES: Standard evaluation system
TF: Transcription factor
TPP: Trehalose-6-phosphate phosphatase
WEGO: Web gene ontology annotation plot
