# Supplementary figures and images for: Genes, pathways and transcription factors involved in seedling stage chilling stress tolerance in indica rice through RNA-Seq analysis
Source: BMC Plant Biol. 2019 Aug 14;19:352. doi: 10.1186/s12870-019-1922-8 (PMC6694648; doi:10.1186/s12870-019-1922-8)

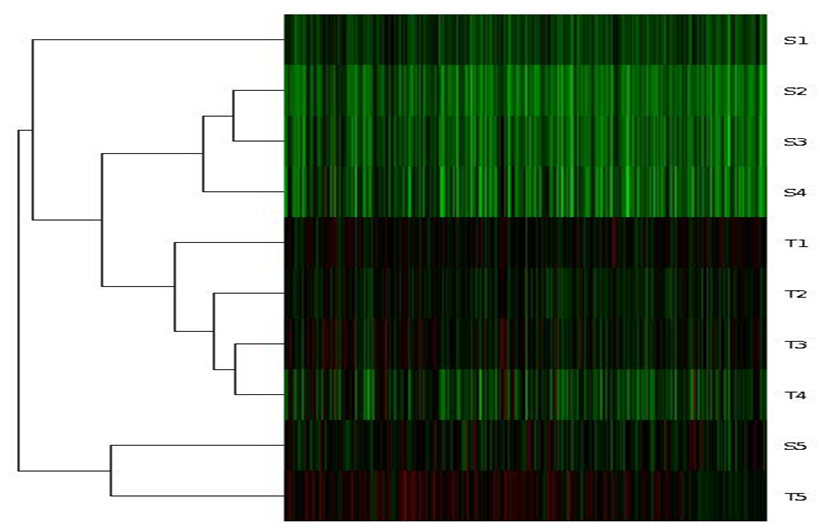

Supplement: Supplementary file 1 — Figure S1. Hierarchical cluster analysis of transcripts in CSV and CTV. The median ratio (stressed/control) was log (base 2)-transformed and subjected to linkage hierarchical clustering. S1-S5 and T1-T5 denote CSV and CTV at 6, 12, 24, 48 and 24 h recovery after 48 h stress conditions, respectively. (TIF 724 kb) [file 12870_2019_1922_MOESM1_ESM.tif]

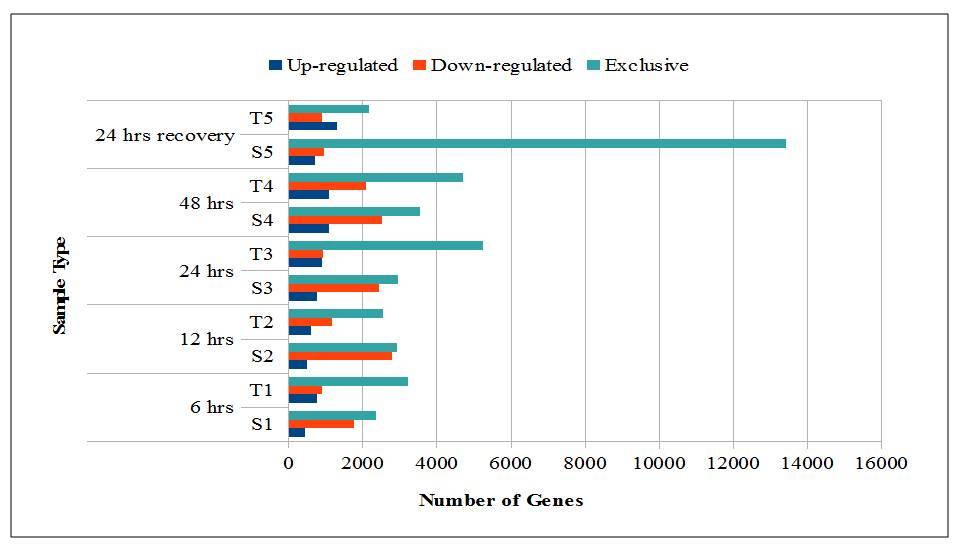

Supplement: Supplementary file 2 — Figure S2. Expression profiles of significant DEGs (up-regulated, down-regulated) and exclusively expressed genes in both genotypes i.e. CSV and CTV. Up and down-regulated genes were identified by comparing the fold change using FPKM values with control condition (S0). T denotes tolerant variety, Geetanjali (CTV) and S denotes susceptible variety, Sahabhagidhan (CSV). (JPG 44 kb) [file 12870_2019_1922_MOESM2_ESM.jpg]

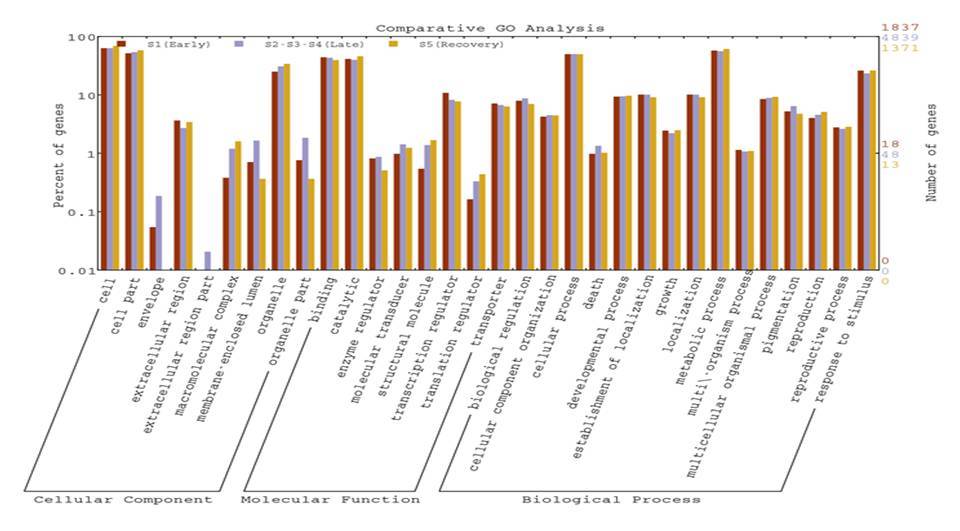

Supplement: Supplementary file 3 — Figure S3. GO analysis of DEGs in early, late and 24 h recovery stages of susceptible cultivar, ‘Sahabhagidhan’. (JPG 62 kb) [file 12870_2019_1922_MOESM3_ESM.jpg]

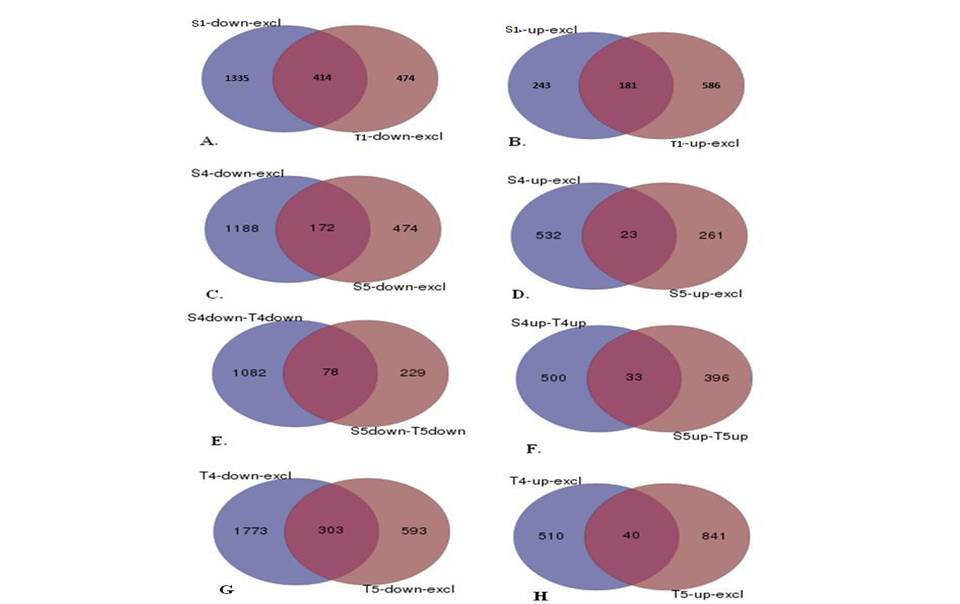

Supplement: Supplementary file 4 — Figure S4. Venn diagram of DEGs under 6 h, 48 h and after 24 h recovery. A) Down-regulated genes of S1 and T1 B) Up-regulated genes of S1 and T1 C) Down-regulated genes of S4 and S5, D) Up-regulated genes of S4 and S5, E) Commonly down-regulated expressed genes of S4-T4 and S5-T5, F) Commonly up-regulated expressed genes of S4-T4 and S5-T5, G) Down-regulated genes of T4 and T5, H) Up-regulated genes of T4 and T5. S, T, 1, 4 and 5 denote CSV, CTV, 6 h, 48 h stress condition and 24 h recovery, respectively. (JPG 33 kb) [file 12870_2019_1922_MOESM4_ESM.jpg]

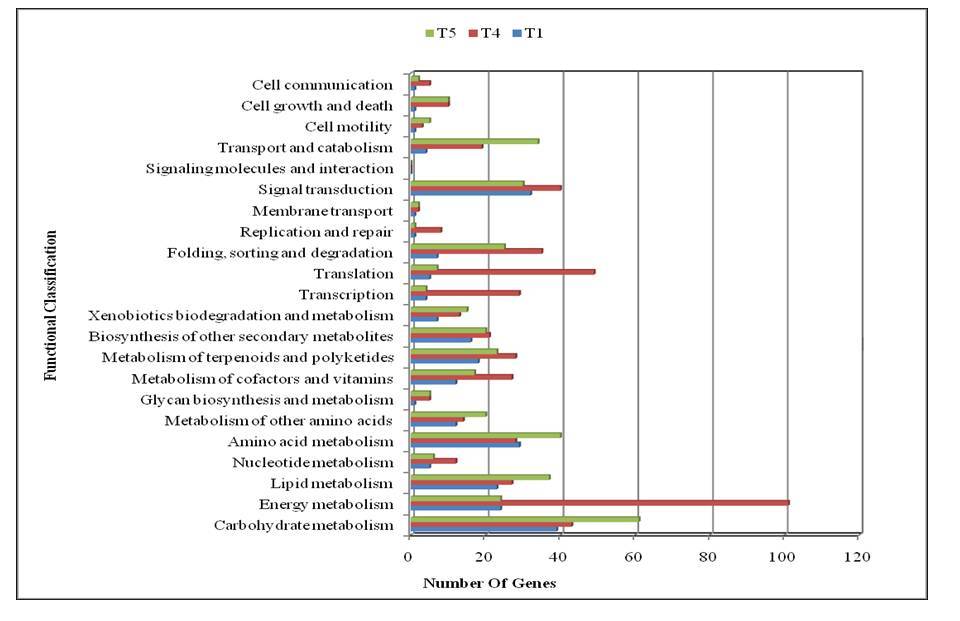

Supplement: Supplementary file 5 — Figure S5. KEGG pathway analysis of DEGs of CSV genotype at 6 h early response phase (S1), 48 h late response phase (S4) and 24 h recovery (S5). (JPG 61 kb) [file 12870_2019_1922_MOESM5_ESM.jpg]

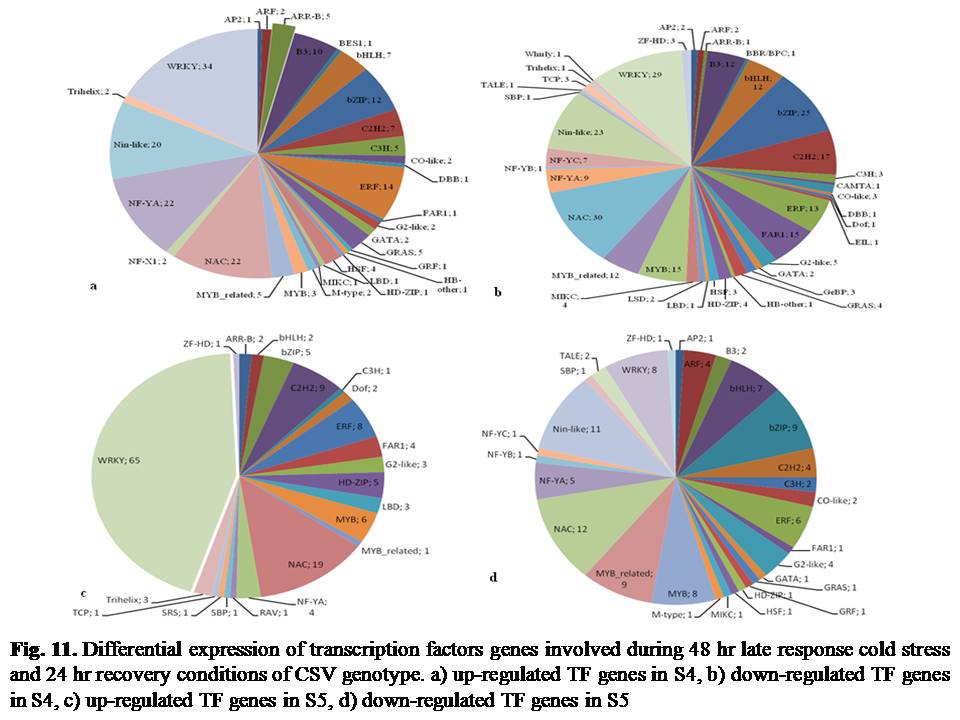

Supplement: Supplementary file 6 — Figure S6. Differential expression of transcription factors genes involved during 48 h late response cold stress and 24 h recovery conditions of CSV genotype. a) up-regulated TF genes in S4, b) down-regulated TF genes in S4, c) up-regulated TF genes in S5, d) down-regulated TF genes in S5. (JPG 82 kb) [file 12870_2019_1922_MOESM6_ESM.jpg]
