# Supplementary material for: Genes, pathways and transcription factors involved in seedling stage chilling stress tolerance in indica rice through RNA-Seq analysis
Source: BMC Plant Biol. 2019 Aug 14;19:352. doi: 10.1186/s12870-019-1922-8 (PMC6694648; doi:10.1186/s12870-019-1922-8)
Supplement: Supplementary file 7 — Table S1. Summary of RNA-Seq reads and their mapping on the rice genome. (DOCX 22 kb) [file 12870_2019_1922_MOESM7_ESM.docx]

**Table S1.** Summary of RNA-Seq reads and their mapping on the rice genome

| **Cold treatment** | | **CSV (cold susceptible variety)** | | | | **CTV (cold tolerant variety)** | | | |
| --- | --- | --- | --- | --- | --- | --- | --- | --- | --- |
| **Hours of cold treatment** | **Temperature (^o^C)** | **Treatments** | **Total reads** | **High quality reads (QV>25)** | **Mapped reads (%)** | **Treatments** | **Total reads** | **High quality reads (QV>25)** | **Mapped reads (%)** |
| 0 | 25 | S0 | 49750582 | 37457861 | 94.48 | T0 | 64525039 | 49484173 | 95.03 |
| 6 | 4 | S1 | 43820375 | 34566321 | 94.54 | T1 | 57035206 | 44104474 | 95.11 |
| 12 | 4 | S2 | 47134484 | 37058762 | 94.87 | T2 | 40161257 | 31480788 | 95.2 |
| 24 | 4 | S3 | 40487255 | 31053565 | 94.05 | T3 | 41608536 | 32606779 | 94.58 |
| 48 | 4 | S4 | 40138395 | 27471998 | 92.42 | T4 | 42292064 | 30020408 | 93.37 |
| 72* | 25 | S5 | 49150400 | 37986658 | 93.28 | T5 | 50263709 | 38577389 | 94.93 |

* The plants subjected to 48h of cold stress were kept at 25**^o^**C for 24h recovery.
