# Supplementary material for: Genes, pathways and transcription factors involved in seedling stage chilling stress tolerance in indica rice through RNA-Seq analysis
Source: BMC Plant Biol. 2019 Aug 14;19:352. doi: 10.1186/s12870-019-1922-8 (PMC6694648; doi:10.1186/s12870-019-1922-8)
Supplement: Supplementary file 8 — Table S2. Differentially expressed genes (DEGs) of CSV and CTV genotype at each time interval. (DOCX 13 kb) [file 12870_2019_1922_MOESM8_ESM.docx]

| **Table S2.** Differentially expressed genes (DEGs) of CSV and CTV genotype at each time interval | | | | | |
| --- | --- | --- | --- | --- | --- |
|  |  |  |  |  |  |
| **Sample** | **Up-regulation** | **Down-regulation** | **DEGs** |  |  |
| S1 | 424 | 1749 | 2173 |  |  |
| S2 | 484 | 2784 | 3268 |  |  |
| S3 | 769 | 2446 | 3215 |  |  |
| S4 | 1088 | 2520 | 3608 |  |  |
| S5 | 713 | 953 | 1666 |  |  |
| **Total** | **3478** | **10452** | **13930** |  |  |
| T1 | 767 | 888 | 1655 |  |  |
| T2 | 594 | 1160 | 1754 |  |  |
| T3 | 901 | 924 | 1825 |  |  |
| T4 | 1083 | 2076 | 3159 |  |  |
| T5 | 1310 | 896 | 2206 |  |  |
| **Total** | **4655** | **5944** | **10599** |  |  |
| **Total_S_T** | **8133** | **16396** | **24529** |  |  |
|  |  |  |  |  |  |
| **Note:** | *1, 2, 3, 4 and 5 denote 6 hrs, 12 hrs, 24 hrs, 48 hrs and recovery after 24 hrs respectively.* | | | | |
|  | *S and T denote for CSV and CTV genotypes respectively.* | | |  |  |
