# Supplementary material for: Genes, pathways and transcription factors involved in seedling stage chilling stress tolerance in indica rice through RNA-Seq analysis
Source: BMC Plant Biol. 2019 Aug 14;19:352. doi: 10.1186/s12870-019-1922-8 (PMC6694648; doi:10.1186/s12870-019-1922-8)
Supplement: Supplementary file 9 — Table S3. Comparison of DEGs of CSV and CTV genotypes at each time interval. (DOCX 14 kb) [file 12870_2019_1922_MOESM9_ESM.docx]

| **Table S3** Comparison of DEGs of CSV and CTV genotypes at each time interval | | | | |  |  |  |
| --- | --- | --- | --- | --- | --- | --- | --- |
|  |  |  |  |  |  |  |  |
| **Treatment time** | **Sample** | **Up-regulation** | **Down-regulation** |  |  |  |  |
| 6 hrs | S1_exclusive | 243 | 1335 |  |  |  |  |
|  | T1_exclusive | 586 | 474 |  |  |  |  |
|  | S1_T1_both | 181 | 414 |  |  |  |  |
| 12 hrs | S2_exclusive | 261 | 2089 |  |  |  |  |
|  | T2_exclusive | 371 | 465 |  |  |  |  |
|  | S2_T2_both | 223 | 695 |  |  |  |  |
| 24 hrs | S3_exclusive | 397 | 1887 |  |  |  |  |
|  | T3_exclusive | 529 | 365 |  |  |  |  |
|  | S3_T3_both | 372 | 559 |  |  |  |  |
| 48 hrs | S4_exclusive | 555 | 1360 |  |  |  |  |
|  | T4_exclusive | 550 | 916 |  |  |  |  |
|  | S4_T4_both | 533 | 1160 |  |  |  |  |
| 24hrs recovery | S5_exclusive | 284 | 646 |  |  |  |  |
|  | T5_exclusive | 881 | 589 |  |  |  |  |
|  | S5_T5_both | 429 | 307 |  |  |  |  |
|  |  |  |  |  |  |  |  |
| **Note:** | *S1_exclusive* | *Exclusively expressed in CSV against CTV at 6 hrs cold stress condition* | | | |  |  |
|  | *T1_exclusive* | *Exclusively expressed in CTV against CSV at 6 hrs cold stress condition* | | | |  |  |
|  | *S1_T1_both* | *Commonly expressed in CSV and CTV at 6 hrs cold stress condition* | | | |  |  |
|  | *S2_exclusive* | *Exclusively expressed in CSV against CTV at 12 hrs cold stress condition* | | | |  |  |
|  | *T2_exclusive* | *Exclusively expressed in CTV against CSV at 12 hrs cold stress condition* | | | |  |  |
|  | *S2_T2_both* | *Commonly expressed in CSV and CTV at 12 hrs cold stress condition* | | | |  |  |
|  | *S3_exclusive* | *Exclusively expressed in CSV against CTV at 24 hrs cold stress condition* | | | |  |  |
|  | *T3_exclusive* | *Exclusively expressed in CTV against CSV at 24 hrs cold stress condition* | | | |  |  |
|  | *S3_T3_both* | *Commonly expressed in CSV and CTV at 24 hrs cold stress condition* | | | |  |  |
|  | *S4_exclusive* | *Exclusively expressed in CSV against CTV at 48 hrs cold stress condition* | | | |  |  |
|  | *T4_exclusive* | *Exclusively expressed in CTV against CSV at 48 hrs cold stress condition* | | | |  |  |
|  | *S4_T4_both* | *Commonly expressed in CSV and CTV at 48 hrs cold stress condition* | | | |  |  |
|  | *S5_exclusive* | *Exclusively expressed in CSV against CTV after 24 hrs recovery condition* | | | |  |  |
|  | *T5_exclusive* | *Exclusively expressed in CTV against CSV after 24 hrs recovery condition at 6 hrs cold stress condition* | | | | | |
|  | *S5_T5_both* | *Commonly expressed in CSV and CTV after 24 hrs recovery condition* | | | |  |  |
|  |  |  |  |  |  |  |  |
