# Supplementary material for: Genes, pathways and transcription factors involved in seedling stage chilling stress tolerance in indica rice through RNA-Seq analysis
Source: BMC Plant Biol. 2019 Aug 14;19:352. doi: 10.1186/s12870-019-1922-8 (PMC6694648; doi:10.1186/s12870-019-1922-8)
Supplement: Supplementary file 13 — Table S7. Significant GO terms enriched DEGs of cold susceptible variety (CSV) on the basis of FDR corrected p-value of three different functional categories. (DOCX 23 kb) [file 12870_2019_1922_MOESM13_ESM.docx]

**Table S7.** Significant GO terms enriched DEGs of cold susceptible variety (CSV) on the basis of FDR corrected p-value of three different functional categories

| **GO term** | **Category** | **Description** | **p-value** | | |
| --- | --- | --- | --- | --- | --- |
|  |  |  | **Early response** | **Late response** | **24hrs recovery** |
| GO:0019748 | Biological process | Secondary metabolic process | 1.10E-010 | 9.50E-006 | 1.50E-009 |
| GO:0050896 | Biological process | Response to stimulus | 3.20E-007 | 1.30E-006 | 7.20E-006 |
| GO:0009719 | Biological process | Response to endogenous stimulus | 1.80E-006 | 3.30E-005 | 0.0022 |
| GO:0006950 | Biological process | Response to stress | 2.80E-005 | 4.00E-006 | 0.0039 |
| GO:0009628 | Biological process | Response to abiotic stimulus | 0.00075 | 0.00042 | 2.60E-006 |
| GO:0009607 | Biological process | Response to biotic stimulus | 1.00E-005 | 0.0037 | NE^*^ |
| GO:0006629 | Biological process | Lipid metabolic process | NE^*^ | NE^*^ | 0.00061 |
| GO:0003700 | Molecular function | Transcription factor activity | 3.10E-009 | 0.00025 | NE |
| GO:0019825 | Molecular function | Oxygen binding | 7.30E-007 | 1.60E-006 | 6.20E-005 |
| GO:0003677 | Molecular function | DNA binding | 6.50E-005 | NE^*^ | NE^*^ |
| GO:0003824 | Molecular function | Catalytic activity | NE^*^ | NE^*^ | 0.00064 |
| GO:0016787 | Molecular function | Hydrolase activity | NE^*^ | NE^*^ | 0.0044 |
| GO:0008289 | Molecular function | Lipid binding | NE^*^ | NE^*^ | 0.0038 |
| GO:0030312 | Cellular component | External encapsulating structure | 2.90E-006 | 5.90E-006 | 0.00035 |
| GO:0005618 | Cellular component | Cell wall | 3.30E-006 | 6.70E-006 | 0.00028 |
| GO:0005576 | Cellular component | Extracellular region | 2.20E-005 | 0.0025 | 0.0013 |
| GO:0009579 | Cellular component | Thylakoid | NE^*^ | NE^*^ | 6.70E-010 |
| GO:0009536 | Cellular component | Plastid | NE^*^ | NE^*^ | 0.00014 |

*NE^*^ denotes significantly not enriched genes*
