# Supplementary material for: Genes, pathways and transcription factors involved in seedling stage chilling stress tolerance in indica rice through RNA-Seq analysis
Source: BMC Plant Biol. 2019 Aug 14;19:352. doi: 10.1186/s12870-019-1922-8 (PMC6694648; doi:10.1186/s12870-019-1922-8)
Supplement: Supplementary file 15 — Table S9. Significant GO terms of late response phase (S2-S4) of CSV genotype. (DOCX 13 kb) [file 12870_2019_1922_MOESM15_ESM.docx]

| **Table S9.** Significant GO terms of late response phase (S2-S4) of CSV genotype | | | |  |  |
| --- | --- | --- | --- | --- | --- |
|  |  |  |  |  |  |
| **GO term** | **Ontology** | **Description** | **Number in input list** | **Number in BG/Ref** | **p-value** |
| GO:0050896 | P | response to stimulus | 1131 | 6928 | 1.30E-006 |
| GO:0006950 | P | response to stress | 774 | 4660 | 4.00E-006 |
| GO:0019748 | P | secondary metabolic process | 121 | 583 | 9.50E-006 |
| GO:0009719 | P | response to endogenous stimulus | 351 | 2015 | 3.30E-005 |
| GO:0009628 | P | response to abiotic stimulus | 495 | 3022 | 0.00042 |
| GO:0009607 | P | response to biotic stimulus | 235 | 1404 | 0.0037 |
| GO:0019825 | F | oxygen binding | 90 | 390 | 1.60E-006 |
| GO:0030528 | F | transcription regulator activity | 398 | 2374 | 0.00025 |
| GO:0003700 | F | transcription factor activity | 398 | 2374 | 0.00025 |
| GO:0030312 | C | external encapsulating structure | 224 | 1189 | 5.90E-006 |
| GO:0005618 | C | cell wall | 222 | 1179 | 6.70E-006 |
| GO:0005576 | C | extracellular region | 131 | 730 | 0.0025 |
| *Note: P, F, C denote for biological process, molecular function and cellular component respectively.* | | | | |  |
