# Supplementary material for: Genes, pathways and transcription factors involved in seedling stage chilling stress tolerance in indica rice through RNA-Seq analysis
Source: BMC Plant Biol. 2019 Aug 14;19:352. doi: 10.1186/s12870-019-1922-8 (PMC6694648; doi:10.1186/s12870-019-1922-8)
Supplement: Supplementary file 16 — Table S10. Significant GO terms of 24 h recovery condition (S5) of CSV genotype. (DOCX 13 kb) [file 12870_2019_1922_MOESM16_ESM.docx]

| **Table S10.** Significant GO terms of 24 hrs recovery condition (S5) of CSV genotype | | | |  |  |
| --- | --- | --- | --- | --- | --- |
|  |  |  |  |  |  |
| **GO term** | **Ontology** | **Description** | **Number in input list** | **Number in BG/Ref** | **p-value** |
| GO:0019748 | P | secondary metabolic process | 57 | 583 | 1.50E-009 |
| GO:0009628 | P | response to abiotic stimulus | 176 | 3022 | 2.60E-006 |
| GO:0050896 | P | response to stimulus | 359 | 6928 | 7.20E-006 |
| GO:0006629 | P | lipid metabolic process | 81 | 1376 | 0.00061 |
| GO:0009719 | P | response to endogenous stimulus | 108 | 2015 | 0.0022 |
| GO:0006950 | P | response to stress | 226 | 4660 | 0.0039 |
| GO:0019825 | F | oxygen binding | 33 | 390 | 6.20E-005 |
| GO:0003824 | F | catalytic activity | 630 | 13508 | 0.00064 |
| GO:0016787 | F | hydrolase activity | 209 | 4293 | 0.0044 |
| GO:0008289 | F | lipid binding | 25 | 346 | 0.0038 |
| GO:0009579 | C | thylakoid | 70 | 772 | 6.70E-010 |
| GO:0009536 | C | plastid | 243 | 4703 | 0.00014 |
| GO:0030312 | C | external encapsulating structure | 73 | 1189 | 0.00035 |
| GO:0005618 | C | cell wall | 73 | 1179 | 0.00028 |
| GO:0005576 | C | extracellular region | 47 | 730 | 0.0013 |
| *Note: P, F, C denote for biological process, molecular function and cellular component respectively.* | | | | |  |
|  |  |  |  |  |  |
