# Supplementary material for: Genes, pathways and transcription factors involved in seedling stage chilling stress tolerance in indica rice through RNA-Seq analysis
Source: BMC Plant Biol. 2019 Aug 14;19:352. doi: 10.1186/s12870-019-1922-8 (PMC6694648; doi:10.1186/s12870-019-1922-8)
Supplement: Supplementary file 17 — Table S11. Significant GO terms of early response phase (T1) of CTV genotype. (DOCX 14 kb) [file 12870_2019_1922_MOESM17_ESM.docx]

| **Table S11.** Significant GO terms of early response phase (T1) of CTV genotype | | |  |  |  |
| --- | --- | --- | --- | --- | --- |
|  |  |  |  |  |  |
| **GO term** | **Ontology** | **Description** | **Number in input list** | **Number in BG/Ref** | **p-value** |
| GO:0050896 | P | response to stimulus | 409 | 6928 | 1.50E-012 |
| GO:0009719 | P | response to endogenous stimulus | 145 | 2015 | 5.50E-011 |
| GO:0006950 | P | response to stress | 279 | 4660 | 4.30E-010 |
| GO:0019748 | P | secondary metabolic process | 53 | 583 | 4.30E-008 |
| GO:0009607 | P | response to biotic stimulus | 97 | 1404 | 3.10E-007 |
| GO:0009628 | P | response to abiotic stimulus | 174 | 3022 | 3.20E-006 |
| GO:0007165 | P | signal transduction | 111 | 1951 | 0.0002 |
| GO:0050794 | P | regulation of cellular process | 111 | 1951 | 0.0002 |
| GO:0065007 | P | biological regulation | 153 | 2871 | 0.00034 |
| GO:0009875 | P | pollen-pistil interaction | 13 | 118 | 0.00086 |
| GO:0050789 | P | regulation of biological process | 111 | 2132 | 0.0035 |
| GO:0019825 | F | oxygen binding | 39 | 390 | 2.10E-007 |
| GO:0030246 | F | carbohydrate binding | 27 | 280 | 2.60E-005 |
| GO:0016740 | F | transferase activity | 261 | 5200 | 0.00026 |
| GO:0030528 | F | transcription regulator activity | 130 | 2374 | 0.0003 |
| GO:0003700 | F | transcription factor activity | 130 | 2374 | 0.0003 |
| GO:0016301 | F | kinase activity | 142 | 2699 | 0.00083 |
| GO:0016772 | F | transferase activity, transferring phosphorus-containing groups | 142 | 2699 | 0.00083 |
| GO:0003824 | F | catalytic activity | 604 | 13508 | 0.006 |
| *Note: P, F, C denote for biological process, molecular function and cellular component respectively.* | | | |  |  |
