# Supplementary material for: Genes, pathways and transcription factors involved in seedling stage chilling stress tolerance in indica rice through RNA-Seq analysis
Source: BMC Plant Biol. 2019 Aug 14;19:352. doi: 10.1186/s12870-019-1922-8 (PMC6694648; doi:10.1186/s12870-019-1922-8)
Supplement: Supplementary file 18 — Table S12. Significant GO terms of late response phase (T2-T4) of CTV genotype. (DOCX 13 kb) [file 12870_2019_1922_MOESM18_ESM.docx]

| **Table S12.** Significant GO terms of late response phase (T2-T4) of CTV genotype | | | |  |  |
| --- | --- | --- | --- | --- | --- |
|  |  |  |  |  |  |
| **GO term** | **Ontology** | **Description** | **Number in input list** | **Number in BG/Ref** | **p-value** |
| GO:0015979 | P | photosynthesis | 81 | 324 | 3.30E-015 |
| GO:0006091 | P | generation of precursor metabolites and energy | 84 | 480 | 1.70E-007 |
| GO:0050896 | P | response to stimulus | 802 | 6928 | 4.10E-006 |
| GO:0006950 | P | response to stress | 546 | 4660 | 2.60E-005 |
| GO:0009607 | P | response to biotic stimulus | 184 | 1404 | 4.80E-005 |
| GO:0009719 | P | response to endogenous stimulus | 252 | 2015 | 6.30E-005 |
| GO:0009628 | P | response to abiotic stimulus | 359 | 3022 | 0.00016 |
| GO:0019825 | F | oxygen binding | 66 | 390 | 9.60E-006 |
| GO:0030528 | F | transcription regulator activity | 279 | 2374 | 0.0012 |
| GO:0003700 | F | transcription factor activity | 279 | 2374 | 0.0012 |
| GO:0005215 | F | transporter activity | 236 | 2021 | 0.0035 |
| *Note: P, F, C denote for biological process, molecular function and cellular component respectively.* | | | | |  |
