# Supplementary material for: Genes, pathways and transcription factors involved in seedling stage chilling stress tolerance in indica rice through RNA-Seq analysis
Source: BMC Plant Biol. 2019 Aug 14;19:352. doi: 10.1186/s12870-019-1922-8 (PMC6694648; doi:10.1186/s12870-019-1922-8)
Supplement: Supplementary file 19 — Table S13. Significant GO terms of 24 h recovery condition (T5) of CTV genotype. (DOCX 13 kb) [file 12870_2019_1922_MOESM19_ESM.docx]

| **Table S13.** Significant GO terms of 24 hrs recovery condition (T5) of CTV genotype | | | |  |  |
| --- | --- | --- | --- | --- | --- |
|  |  |  |  |  |  |
| **GO term** | **Ontology** | **Description** | **Number in input list** | **Number in BG/Ref** | **p-value** |
| GO:0050896 | P | response to stimulus | 523 | 6928 | 4.30E-011 |
| GO:0006950 | P | response to stress | 355 | 4660 | 7.70E-009 |
| GO:0009628 | P | response to abiotic stimulus | 236 | 3022 | 2.20E-007 |
| GO:0009607 | P | response to biotic stimulus | 123 | 1404 | 6.10E-007 |
| GO:0009719 | P | response to endogenous stimulus | 153 | 2015 | 7.00E-005 |
| GO:0019748 | P | secondary metabolic process | 55 | 583 | 9.20E-005 |
| GO:0006629 | P | lipid metabolic process | 106 | 1376 | 0.00044 |
| GO:0005975 | P | carbohydrate metabolic process | 110 | 1439 | 0.00047 |
| GO:0019825 | F | oxygen binding | 56 | 390 | 8.90E-011 |
| GO:0030528 | F | transcription regulator activity | 179 | 2374 | 3.00E-005 |
| GO:0003700 | F | transcription factor activity | 179 | 2374 | 3.00E-005 |
| GO:0003824 | F | catalytic activity | 855 | 13508 | 0.00017 |
| GO:0016787 | F | hydrolase activity | 287 | 4293 | 0.00082 |
| GO:0030312 | C | external encapsulating structure | 90 | 1189 | 0.0018 |
| GO:0005618 | C | cell wall | 90 | 1179 | 0.0014 |
| GO:0005576 | C | extracellular region | 58 | 730 | 0.0037 |
| *Note: P, F, C denote for biological process, molecular function and cellular component respectively.* | | | | |  |
|  |  |  |  |  |  |
